# Investigating the influence of perinatal nicotine and alcohol exposure on the genetic profiles of dopaminergic neurons in the VTA using miRNA–mRNA analysis

**DOI:** 10.1038/s41598-020-71875-1

**Published:** 2020-09-14

**Authors:** Tina Kazemi, Shuyan Huang, Naze G. Avci, Charlotte Mae K. Waits, Yasemin M. Akay, Metin Akay

**Affiliations:** Department of Biomedical Engineering, University of Houston, Houston, TX 77204 USA

**Keywords:** Neuroscience, Molecular neuroscience, Neurodevelopmental disorders

## Abstract

Nicotine and alcohol are two of the most commonly used and abused recreational drugs, are often used simultaneously, and have been linked to significant health hazards. Furthermore, patients diagnosed with dependence on one drug are highly likely to be dependent on the other. Several studies have shown the effects of each drug independently on gene expression within many brain regions, including the ventral tegmental area (VTA). Dopaminergic (DA) neurons of the dopamine reward pathway originate from the VTA, which is believed to be central to the mechanism of addiction and drug reinforcement. Using a well-established rat model for both nicotine and alcohol perinatal exposure, we investigated miRNA and mRNA expression of dopaminergic (DA) neurons of the VTA in rat pups following perinatal alcohol and joint nicotine–alcohol exposure. Microarray analysis was then used to profile the differential expression of both miRNAs and mRNAs from DA neurons of each treatment group to further explore the altered genes and related biological pathways modulated. Predicted and validated miRNA-gene target pairs were analyzed to further understand the roles of miRNAs within these networks following each treatment, along with their post transcription regulation points affecting gene expression throughout development. This study suggested that glutamatergic synapse and axon guidance pathways were specifically enriched and many miRNAs and genes were significantly altered following alcohol or nicotine–alcohol perinatal exposure when compared to saline control. These results provide more detailed insight into the cell proliferation, neuronal migration, neuronal axon guidance during the infancy in rats in response to perinatal alcohol/ or nicotine–alcohol exposure.

## Introduction

Alcohol and cigarettes are among the most commonly used and abused legal drugs. Studies have shown people who smoke are much more likely to drink, and vice versa^1–5^. Additionally, individuals with high recreational alcohol use tend to smoke cigarettes at higher rates than the general population, suggesting this link between drinking and smoking may also be dose-dependent^4,6,7^. Of particular importance, maternal smoking and/or drinking during pregnancy increase the risk of health problems for the developing baby. Nicotine alters the chemistry in the developing brain, which have been associated with developmental, cognitive, and behavioral deficits including preterm birth, low birth weight, birth defects, learning disabilities, attention deficit hyperactivity disorder (ADHD), and drug use and abuse later in life^8–12^. Additionally, alcohol can cross the placenta and has been linked to dysfunctional regulation of several neurotransmitters^13^. Specifically, perinatal alcohol exposure has been linked to birth complications and developmental disabilities, including fetal alcohol spectrum disorders (FASDs), stillbirth, preterm (early) birth, along with many cognition and behavior problems later in life ^14^. The Centers for Disease Control and Prevention (CDC) reports that 1 in 14 women who gave birth in the United States in the year 2016 (7.2%) smoked cigarettes during pregnancy^15^. Additionally, the CDC reported in the 2015–2017 timeline, drinking and binge drinking by pregnant women was 11.5% and 3.9%, respectively^16^.

Addictive substances act on the brain’s reward system by triggering dopamine (DA) release through the activation of the mesocorticolimbic DA system, also known as the reward circuitry in the brain. In the mesocorticolibic pathway, DA neurons originating from the ventral tegmental area (VTA) project to the striatum, prefrontal cortex (PFC), and the nucleus accumbens (NAc)^17^. This pathway/system mediates the reinforcing and/or withdrawal properties of addictive substances^17–19^. Although alcohol has a wider range of molecular targets than nicotine, both drugs exert their reinforcing properties through the activation of the mesocorticolimbic system, leading to increased DA transmission, which is thought to be integral to their ability to cause dependence.

Nicotine has been identified as the biologically active and addictive component in tobacco^20^. Studies have shown that the common use of nicotine can enhance intellectual performance, decrease depression and anxiety, and activate the DA reward system^21–23^. In adults, nicotine exposure induces neurotransmitter function through the stimulation of DA neurons in the VTA, which mediates the release of DA and causes increased neuronal firing along the projection pathways^24^. The systemic nicotine exposures, including both daily intravenous nicotine injection^25,26^ and a subcutaneously implanted osmotic minipump^27–29^ are responsible for enhancing DA release within the NAc through the stimulation of VTA DA neurons. Nicotine is transmitted from the pregnant mother to the offspring through the placenta during pregnancy and through the breast milk after birth. Studies have shown persistent gene alterations in brain regions involved in the reward pathway and neurodevelopmental changes at the cellular level caused by both prenatal nicotine exposure^30–34^ and subsequent 14 days of nicotine exposure after birth^29^. Perinatal nicotine exposure is also associated with learning disabilities, cognitive dysfunction, and can indicate higher risk of psychiatric problems such as substance abuse later in life^33,35^.

Alcohol acts as a depressant by altering the balance between inhibitory and excitatory neurotransmission by increasing the inhibitory neurotransmission in the brain. Alcohol consumption is accompanied by decreased attention, alterations in memory, mood changes, and drowsiness. Its continued consumption can result in lethargy, confusion, amnesia, loss of sensation, difficulty in breathing, and even death^36^. Alcohol crosses from mother’s bloodstream through the placenta and directly enters into the unborn baby’s bloodstream, directly impacting the embryonic development of the fetus. Alcohol exposure throughout and after pregnancy over a period of 3‐trimester gestational exposure results in dysfunctional regulation of several neurotransmitters, including serotonin, glutamate, noradrenaline, acetylcholine, histamine, and dopamine^13,28,37,38^. Perinatal substance abuse does not only impact fetal brain development, resulting in behavioral disorders but also adult offspring exposed to nicotine during gestation self‐administer significantly more nicotine^28,39,40^.

MicroRNAs (miRNA) have been recently used to study addiction by serving as popular biomarkers^41^. miRNAs are highly conserved, non-coding RNA sequences that bind to target sites within the 3′ untranslated regions in target messenger RNAs (mRNA) to regulate their stability and translation. In the brain, the interaction between miRNAs and their target mRNAs are thought to modulate the developmental processes like neurogenesis and neural differentiation and contribute to synaptic plasticity^42–44^. Studies have examined the interaction of miRNAs and downstream gene expression to study the regulatory pathways linked to addiction, drug use and abuse, as well as their developmental and long-term effects, specifically within brain reward pathways^41,45–47^. Given the importance of the VTA in addiction, elucidating the potential of miRNAs’ influence on gene expression in addiction could be a very useful tool.

We have recently investigated the influence of perinatal nicotine exposure on genetic expression profiles of the dopaminergic neurons in the VTA^31^. Our study suggested dopaminergic synapse pathway, nicotine addiction, as well as neurotrophin signaling pathway to be significantly altered in rat pups perinatally exposed to nicotine. The expression of several miRNAs and genes were altered suggesting involvement of many biological pathways. Considering that nicotine and alcohol are often used simultaneously, in this study we further investigated the transcriptional and post-transcriptional gene regulation modulations following exposure to alcohol or combined nicotine–alcohol during gestational developmental stages. In order to better understand on a molecular level, the mechanisms underlying gestational exposure to alcohol and nicotine–alcohol during neurodevelopment, differentially expressed miRNA and mRNA from DA neurons of the VTA at the single cell level were investigated. Fluorescent activated cell sorting (FACS) methods were used to collect DA neurons from the VTA brain slices, prior to microarray expression analysis. Finally, miRNA–mRNA validated and predicted target pairs were identified and analyzed using MultimiR to better understand perinatal alcohol or nicotine–alcohol exposure and its downstream effect on the miRNA-gene pairs. Database for Annotation, Visualization and Integrated Discovery (DAVID) was used to perform pathway enrichment analysis in order to interpret the function of our differentially expressed gene (DEG) list following each treatment group.

## Results

miRNA and gene expression profiling were done on VTA DA neuron samples collected from “alcohol”, combined “nicotine–alcohol”, and “saline” (control) perinatally treated pups from gestational day (GD6) to postnatal day (PND 10–14), which is developmentally equivalent to the three trimesters of human pregnancy^48–50^. Samples were isolated, dissociated and sorted (see “Materials and Methods”). Fixed intact cells that were double stained for both NeuN (neuronal nuclei antibody) and tyrosine hydroxylase (TH) antibodies were collected using FACS. Total RNA and miRNAs were extracted and samples were processed using Agilent Sureprint miRNA and mRNA microarrays, respectively in order to compare miRNA and gene expression profiles between treatment groups.

### miRNA and mRNA expression analysis following perinatal alcohol and nicotine–alcohol exposure

**Figure 1:**
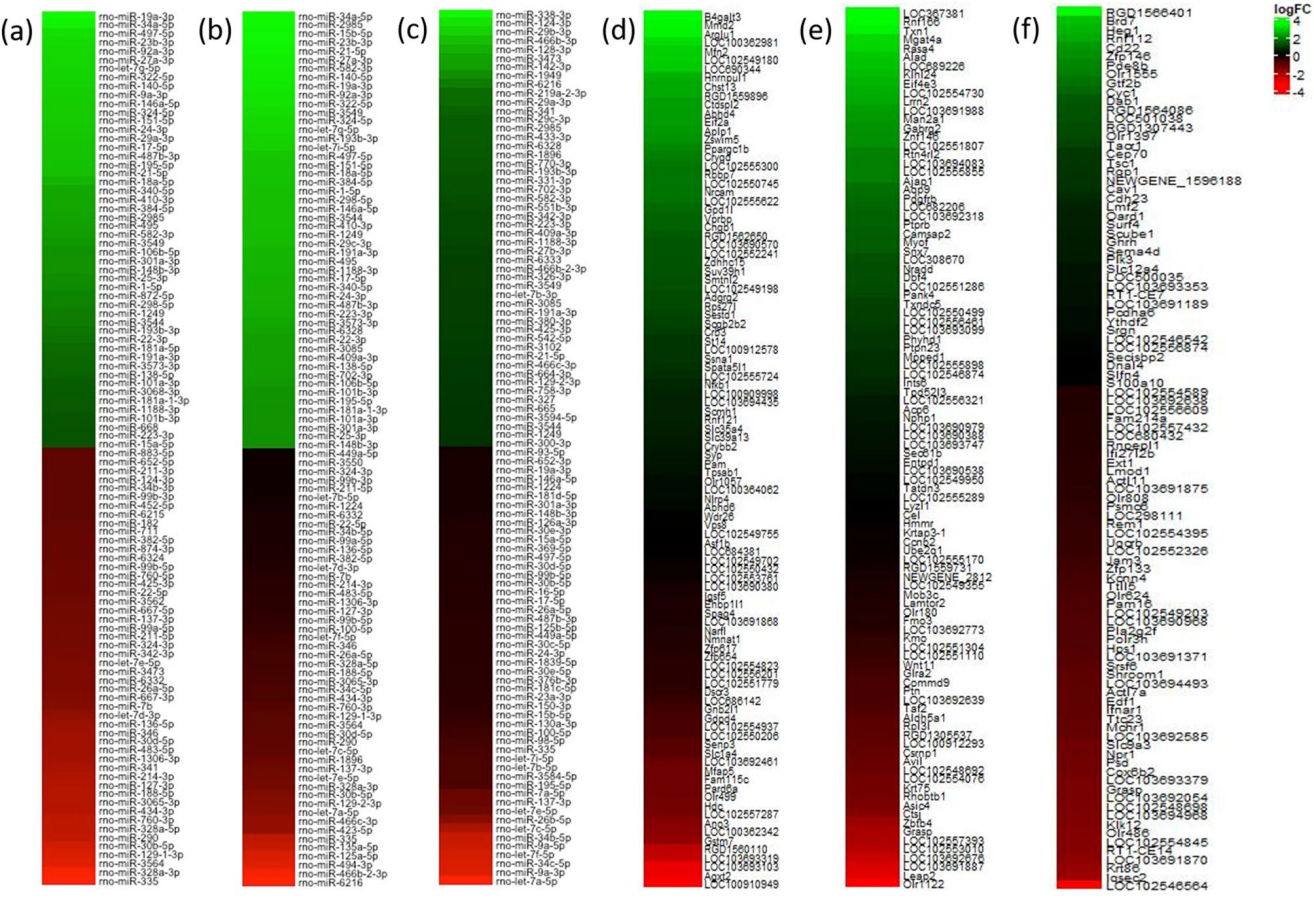
Heat maps of the DEmiRs and DEGs. Top 50 DEmiRs in DA neurons of the VTA following perinatal (**a**) alcohol exposure compared to saline control, (**b**) nicotine–alcohol exposure compared to saline control, and (**c**) nicotine–alcohol exposure compared to alcohol exposure. Top 50 DEGs in DA neurons of the VTA following perinatal (**d**) alcohol exposure compared to saline control, (**e**) nicotine–alcohol exposure compared to saline control, and (**f**) nicotine–alcohol exposure compared to alcohol exposure. Expression profiles are based on greatest absolute log fold change.

Differential expression among genes was calculated for VTA DA neurons for both alcohol and nicotine–alcohol treatment groups by contrasting each group to the saline control group. The nicotine–alcohol treatment group was also contrasted against the alcohol group for an additional comparison. Following alcohol treatment, 1,257 unique genes were found to be differentially upregulated and 330 were differentially downregulated. Following perinatal nicotine–alcohol treatment, 1,771 genes were upregulated and 269 were downregulated. Following perinatal nicotine–alcohol treatment contrasted against the alcohol group, 2,113 genes were upregulated and 1,836 were downregulated. Statistical analysis was done using Benjamini and Hochberg (BH) method with q value < 0.05 and an absolute log2 fold change > 1 as previously described in Keller et al.^31,32^. Figure 1 illustrates the heatmaps of the top 50 significantly differentially expressed miRNAs (DEmiRs) following perinatal (a) alcohol and (b) nicotine–alcohol exposures compared to the saline control group, and (c) nicotine–alcohol exposure compared to the alcohol treatment group. Top 50 significantly DEGs is shown in Fig. 1 following perinatal (d) alcohol and (e) nicotine–alcohol exposures compared to the saline control group, and (f) nicotine–alcohol exposure compared to the alcohol treatment group. Table 1 shows the details of top 20 significantly up and downregulated DEmiRs, which further target genes in DA neurons of the VTA following (a) perinatal alcohol, (b) perinatal nicotine–alcohol, and (c) perinatal nicotine–alcohol vs. alcohol exposures.

**Table 1:** Top 20 significantly differentially expressed miRNAs.

miRNA accession	miRNA name	Log FC	adj p val	miRNA accession	miRNA name	Log FC	adj p val
Upregulated				Downregulated			
**(a) Perinatal alcohol exposure**							
MIMAT0004646	rno-miR-338-3p	2.204	1.91E−04	MIMAT0024856	rno-miR-6216	− 0.984	4.53E−05
MIMAT0000847	rno-miR-142-3p	0.631	1.91E−04	MIMAT0000829	rno-miR-125a-5p	− 0.712	1.78E−04
MIMAT0017852	rno-miR-1949	0.944	2.27E−04	MIMAT0005278	rno-miR-466b-5p	− 1.050	2.27E−04
MIMAT0005446	rno-miR-219a-2-3p	1.448	2.27E−04	MIMAT0000606	rno-miR-7a-5p	− 0.710	2.36E−04
MIMAT0000602	rno-miR-20a-5p	0.832	2.27E−04	MIMAT0000804	rno-miR-30c-5p	− 0.741	2.74E−04
MIMAT0000788	rno-miR-19b-3p	0.783	2.44E−04	MIMAT0003193	rno-miR-494-3p	− 0.739	4.19E−04
MIMAT0000889	rno-miR-219a-5p	1.440	2.74E−04	MIMAT0000830	rno-miR-125b-5p	− 0.966	4.19E−04
MIMAT0000785	rno-miR-16-5p	0.644	4.19E−04	MIMAT0035732	rno-miR-1896	− 0.544	4.19E−04
MIMAT0003211	rno-miR-20b-5p	0.592	5.79E−04	MIMAT0000841	rno-miR-135a-5p	− 0.520	5.79E−04
MIMAT0000784	rno-miR-15b-5p	0.579	1.15E−03	MIMAT0017120	rno-miR-129-1-3p	− 0.404	1.41E−03
MIMAT0000798	rno-miR-27b-3p	0.627	1.16E−03	MIMAT0000601	rno-miR-129-2-3p	− 0.550	1.41E−03
MIMAT0000816	rno-miR-92a-3p	0.424	1.96E−03	MIMAT0000885	rno-miR-214-3p	− 0.317	1.41E−03
MIMAT0000801	rno-miR-29b-3p	0.542	2.48E−03	MIMAT0017029	rno-miR-328a-5p	− 0.351	2.34E−03
MIMAT0000794	rno-miR-24-3p	0.391	3.20E−03	MIMAT0005315	rno-miR-434-3p	− 0.331	3.46E−03
MIMAT0000799	rno-miR-27a-3p	0.417	3.84E−03	MIMAT0000806	rno-miR-30b-5p	− 0.387	3.47E−03
MIMAT0000779	rno-let-7i-5p	0.509	4.38E−03	MIMAT0000575	rno-miR-335	− 0.473	4.38E−03
MIMAT0000793	rno-miR-23b-3p	0.428	4.38E−03	MIMAT0005301	rno-miR-188-5p	− 0.330	4.54E−03
MIMAT0000789	rno-miR-19a-3p	0.494	4.54E−03	MIMAT0017305	rno-miR-423-5p	− 0.554	4.65E−03
MIMAT0003200	rno-miR-487b-3p	0.382	5.67E−03	MIMAT0017286	rno-miR-466b-2-3p	− 0.865	5.99E−03
MIMAT0000815	rno-miR-34a-5p	0.471	6.80E−03	MIMAT0017287	rno-miR-466c-3p	− 0.594	1.03E−02
**(b) Perinatal nicotine–alcohol exposure**							
MIMAT0000848	rno-miR-142-3p	0.885	5.54E−05	MIMAT0024856	rno-miR-6216	− 0.794	5.54E−05
MIMAT0000581	rno-miR-338-3p	2.557	5.54E−05	MIMAT0000606	rno-miR-7a-5p	− 0.936	5.54E−05
MIMAT0017852	rno-miR-1949	1.174	5.54E−05	MIMAT0000829	rno-miR-125a-5p	− 0.704	5.54E−05
MIMAT0000889	rno-miR-219a-5p	1.938	5.54E−05	MIMAT0000804	rno-miR-30c-5p	− 0.842	1.34E−04
MIMAT0005446	rno-miR-219a-2-3p	1.617	8.68E−05	MIMAT0005278	rno-miR-466b-5p	− 1.065	1.34E−04
MIMAT0000602	rno-miR-20a-5p	0.827	1.59E−04	MIMAT0000841	rno-miR-135a-5p	− 0.645	1.59E−04
MIMAT0000801	rno-miR-29b-3p	0.886	1.59E−04	MIMAT0000830	rno-miR-125b-5p	− 1.086	1.86E−04
MIMAT0000788	rno-miR-19b-3p	0.775	1.86E−04	MIMAT0003193	rno-miR-494-3p	− 0.712	4.99E−04
MIMAT0000798	rno-miR-27b-3p	0.713	5.43E−04	MIMAT0000575	rno-miR-335	− 0.630	1.01E−03
MIMAT0003211	rno-miR-20b-5p	0.558	8.79E−04	MIMAT0000806	rno-miR-30b-5p	− 0.479	1.11E−03
MIMAT0000785	rno-miR-16-5p	0.545	9.76E−04	MIMAT0035732	rno-miR-1896	− 0.414	1.99E−03
MIMAT0000802	rno-miR-29a-3p	0.544	1.81E−03	MIMAT0017120	rno-miR-129-1-3p	− 0.381	1.99E−03
MIMAT0035734	rno-miR-193b-3p	0.367	2.14E−03	MIMAT0005315	rno-miR-434-3p	− 0.348	2.50E−03
MIMAT0000816	rno-miR-92a-3p	0.411	2.23E−03	MIMAT0000601	rno-miR-129-2-3p	− 0.484	2.57E−03
MIMAT0017360	rno-miR-582-3p	0.432	2.43E−03	MIMAT0000781	rno-miR-9a-5p	− 1.190	2.57E−03
MIMAT0000799	rno-miR-27a-3p	0.440	2.59E−03	MIMAT0017029	rno-miR-328a-5p	− 0.324	2.93E−03
MIMAT0017807	rno-miR-3549	0.392	2.70E−03	MIMAT0000807	rno-miR-30d-5p	− 0.388	3.38E−03
MIMAT0000793	rno-miR-23b-3p	0.456	2.86E−03	MIMAT0000885	rno-miR-214-3p	− 0.257	3.59E−03
MIMAT0000900	rno-miR-298-5p	0.318	2.93E−03	MIMAT0000821	rno-miR-99b-5p	− 0.283	3.61E−03
MIMAT0000784	rno-miR-15b-5p	0.460	2.93E−03	MIMAT0005301	rno-miR-188-5p	− 0.332	3.75E−03

miRNA accession	miRNA name	Log FC	adj p val				
**(c) Perinatal nicotine–alcohol exposure vs. alcohol exposure**							
Upregulated							
MIMAT0000276	rno-miR-219a-5p	0.504	0.277				
MIMAT0000100	rno-miR-29b-3p	0.334	0.361				
MIMAT0000434	rno-miR-142-3p	0.243	0.462				
MIMAT0000422	rno-miR-124-3p	0.358	0.462				
	rno-miR-6216	0.183	0.620				
Downregulated							
MIMAT0000685	rno-miR-34b-5p	− 0.370	0.108				
MIMAT0000686	rno-miR-34c-5p	− 0.399	0.108				
MIMAT0000083	rno-miR-26b-5p	− 0.261	0.277				
MIMAT0000606	rno-miR-7a-5p	− 0.207	0.277				
MIMAT0000774	rno-let-7a-5p	− 0.465	0.399				
MIMAT0000778	rno-let-7f.-5p	− 0.394	0.462				
MIMAT0000098	rno-miR-100-5p	− 0.138	0.462				
MIMAT0000429	rno-miR-137-3p	− 0.207	0.999				

(DEmiRs) in DA neurons of the VTA following perinatal (a) alcohol exposure compared to saline control, (b) nicotine-alcohol compared to saline control, and (c) nicotine-alcohol compared to alcohol exposure. Benjamini–Hochberg method was used for the statistical analysis (q value < 0.05, absolute log2 fold change > 0.5).

Following alcohol exposure, 51 miRNAs were differentially upregulated and 39 were differentially downregulated. Following combined nicotine–alcohol exposure, 51 miRNAs were differentially upregulated and 41 were differentially downregulated. Following nicotine–alcohol exposure 5 miRNAs were upregulated and 8 were downregulated when compared to the alcohol treatment group. The BH method was used for the statistical analysis applying parameters of q value < 0.05 and an absolute log2 fold change > 0.5 as previously described in Keller et al.^31,32^. Table 2a–c list the most significant up and downregulated DEGs along with their description and predicted miRNA targets for perinatal alcohol, nicotine–alcohol and nicotine–alcohol vs. alcohol treatment groups, respectively.

**Table 2:** Top DEGs from microarray expression analysis of DA neurons.

Gene Symbol	Entrez ID	Log FC	adj p val	Description	miRNA target
**(a) Perinatal alcohol exposure**					
Upregulated					
Lypla2	83510	9.153	1.07E−05	lysophospholipase 2	rno-miR-125a-5p rno-miR-125b-5p rno-miR-434-3p rno-miR-6332
Tprg1l	687090	9.986	1.07E−05	tumor protein p63 regulated 1 like	
LOC103689999	103689999	9.070	1.07E−05	saccharopine dehydrogenase-like oxidoreductase	
LOC103690032	103690032	9.856	1.07E−05	insulinoma-associated protein 1-like	
Gnai2	81664	7.971	1.07E−05	G protein subunit alpha i2	rno-miR-129-1-3p rno-miR-129-2-3p rno-miR-30b-5p rno-miR-30d-5p
Psmc5	81827	7.531	1.07E−05	proteasome 26S subunit, ATPase 5	
Ano10	301111	7.514	1.07E−05	anoctamin 10	
Uba1	314432	6.827	1.15E−05	ubiquitin-like modifier activating enzyme 1	
Bnip3l	140923	7.468	1.17E−05	BCL2 interacting protein 3 like	rno-miR-106b-5p rno-miR-20a-5p rno-miR-23b-3p rno-miR-27a-3p rno-miR-27b-3p rno-miR-384-5p rno-miR-129-1-3p rno-miR-129-2-3p rno-miR-30b-5p rno-miR-30d-5p
Rpl4	64302	6.871	1.21E−05	ribosomal protein L4	
Atp5i	140608	7.906	1.23E−05	ATP synthase membrane subunit e	rno-miR-324-3p
Derl1	362912	6.542	1.23E−05	derlin 1	rno-miR-466b-2-3p
Cpsf7	365407	7.130	1.23E−05	cleavage and polyadenylation specific factor 7	rno-miR-711
LOC498154	498154	8.000	1.23E−05	hypothetical protein LOC498154	
Cotl1	361422	6.275	1.23E−05	coactosin-like F-actin binding protein 1	rno-miR-135a-5p rno-miR-30b-5p rno-miR-30d-5p rno-miR-760-3p
Atp9a	84011	6.550	1.23E−05	ATPase phospholipid transporting 9A	
Atp5g1	29754	6.185	1.23E−05	ATP synthase membrane subunit c locus 1	rno-miR-214-3p rno-miR-3065-3p
Cdc37	114562	7.369	1.24E−05	cell division cycle 37	
Downregulated					
RGD1564541	313433	− 6.140	1.41E−05	hypothetical protein FLJ22965	rno-miR-106b-5p rno-miR-17-5p rno-miR-20a-5p rno-miR-20b-5p
Coprs	290925	− 7.360	1.77E−05	coordinator of PRMT5 and differentiation stimulator	rno-miR-340-5p
LOC102546787	102546787	− 5.790	3.63E−05	uncharacterized LOC102546787	
LOC102552625	102552625	− 6.179	4.66E−05	uncharacterized LOC102552625	
LOC103693454	103693454	− 7.092	5.37E−05	uncharacterized LOC103693454	
Hint2	313491	− 4.330	6.76E−05	histidine triad nucleotide binding protein 2	
Rab33b	365793	− 4.538	6.76E−05	member RAS oncogene family	rno-miR-19a-3p
LOC102551839	102551839	− 5.450	7.27E−05	uncharacterized LOC102551839	
Got2	25721	− 4.102	8.36E−05	glutamic-oxaloacetic transaminase 2	rno-miR-384-5p
Vav2	296603	− 3.635	8.89E−05	vav guanine nucleotide exchange factor 2	rno-miR-15b-5p rno-miR-16-5p rno-miR-195-5p rno-miR-19a-3p rno-miR-27a-3p rno-miR-27b-3p rno-miR-29a-3p rno-miR-322-5p rno-miR-497-5p
LOC103690624	103690624	− 5.005	9.58E−05	uncharacterized LOC103690624	
**(b) Perinatal nicotine–alcohol exposure**					
Upregulated					
Lypla2	83510	9.585	6.92E−06	lysophospholipase 2	rno-miR-125a-5p rno-miR-125b-5p rno-miR-434-3p rno-miR-6332
LOC103689999	103689999	9.378	7.03E−06	saccharopine dehydrogenase-like oxidoreductase	
Tprg1l	687090	9.872	7.03E−06	tumor protein p63 regulated 1 like	
Psmc5	81827	7.906	7.03E−06	proteasome 26S subunit, ATPase 5	
Ssbp4	364534	7.510	7.03E−06	single stranded DNA binding protein 4	rno-miR-26a-5p
LOC103690032	103690032	9.960	7.03E−06	uncharacterized LOC103690032	
Gnai2	81664	7.999	7.57E−06	G protein subunit alpha i2	rno-miR-129-1-3p rno-miR-129-2-3p rno-miR-30b-5p rno-miR-30d-5p
Atp9a	84011	7.417	7.62E−06	ATPase phospholipid transporting 9A	
Uba1	314432	7.100	7.62E−06	ubiquitin-like modifier activating enzyme 1	
Ano10	301111	7.457	7.62E−06	anoctamin 10	
Atp5g1	29754	6.851	7.62E−06	ATP synthase membrane subunit c locus 1	rno-miR-214-3p rno-miR-3065-3p
Bnip3l	140923	7.773	8.15E−06	BCL2 interacting protein 3 like	rno-miR-129-1-3p rno-miR-129-2-3p rno-miR-137-3p rno-miR-30b-5p rno-miR-30d-5p rno-miR-20a-5p rno-miR-23b-3p rno-miR-27a-3p rno-miR-27b-3p rno-miR-384-5p rno-miR-26a-5p
Derl1	362912	6.952	8.45E−06	derlin 1	rno-miR-466b-2-3p rno-miR-26a-5p
Sema4a	310630	7.562	8.48E−06	semaphorin 4A	
Npdc1	296562	6.914	9.31E−06	neural proliferation, differentiation and control, 1	rno-miR-760-3p
LOC498154	498154	8.113	9.31E−06	hypothetical protein LOC498154	rno-let-7e-5p
Vamp3	29528	7.066	9.31E−06	vesicle-associated membrane protein 3	rno-miR-34c-5p
Cdc37	114562	7.642	9.31E−06	cell division cycle 37	rno-miR-99b-5p
Atp5i	140608	7.934	9.31E−06	ATP synthase membrane subunit e	rno-miR-324-3p
LOC102546798	102546798	9.532	9.31E−06	uncharacterized LOC102546798	
Downregulated					
RGD1564541	313433	− 6.121	1.10E−05	similar to hypothetical protein FLJ22965	rno-miR-20a-5p rno-miR-20b-5p rno-miR-17-5p
Coprs	290925	− 7.780	1.26E−05	coordinator of PRMT5 and differentiation stimulator	rno-miR-340-5p
Hint2	313491	− 4.669	4.28E−05	histidine triad nucleotide binding protein 2	
LOC102546787	102546787	− 5.142	5.94E−05	uncharacterized LOC102546787	
Rab33b	365793	− 4.539	6.32E−05	member RAS oncogene family	rno-miR-19a-3p
Snrpd3	687711	− 4.206	7.25E−05	small nuclear ribonucleoprotein D3 polypeptide	
Ufd1l	84478	− 5.237	7.64E−05	ubiquitin recognition factor in ER associated degradation 1	
LOC102551839	102551839	− 5.315	7.82E−05	uncharacterized LOC102551839	
Cr1l	54243	− 5.646	8.74E−05	complement C3b/C4b receptor 1 like	rno-miR-15b-5p
**(c) Perinatal nicotine–alcohol exposure vs. alcohol exposure**					
Upregulated					
Gnl3	290,556	3.598	5.13E−05	guanine nucleotide binding protein-like 3 (nucleolar)	
Usp34	360,990	3.908	5.60E−05	ubiquitin specific peptidase 34	
Fam91a1	689,997	3.925	9.43E−05	family with sequence similarity 91	
Bbs5	362,142	3.507	9.57E−05	Bardet-Biedl syndrome 5	
RGD1566401	500,717	3.991	9.66E−05	similar to GTL2	
Rars	287,191	3.882	1.25E−04	arginyl-tRNA synthetase	
Hmbox1	305,968	3.757	1.25E−04	homeobox containing 1	rno-miR-137-3p rno-miR-34c-5p rno-miR-34b-5p
Kidins220	116,478	3.949	1.32E−04	kinase D-interacting substrate 220	rno-miR-34c-5p rno-miR-34b-5p
Ptbp2	310,820	3.703	1.38E−04	polypyrimidine tract binding protein 2	
Tubg2	680,991	3.542	1.51E−04	tubulin	
Ccdc50	288,022	3.577	1.52E−04	coiled-coil domain containing 50	rno-miR-137-3p
Impdh1	362,329	3.475	1.54E−04	IMP (inosine 5′-monophosphate) dehydrogenase 1	rno-miR-100-5p
Ppwd1	294,711	4.105	1.63E−04	peptidylprolyl isomerase domain and WD repeat containing 1	
Rdx	315,655	3.776	1.78E−04	radixin	rno-let-7a-5p rno-let-7f.-5p
Rapgef2	310,533	3.749	1.85E−04	Rap guanine nucleotide exchange factor (GEF) 2	
RGD1566401	500,717	4.177	1.89E−04	similar to GTL2	
Cacna1b	257,648	3.542	1.98E−04	calcium channel	
Cst3	25,307	3.970	1.99E−04	cystatin C	
Ubxn2b	312,965	3.712	2.16E−04	UBX domain protein 2B	
Slc7a3	29,485	3.661	2.23E−04	solute carrier family 7 (cationic amino acid transporter	
Cdc42	64,465	3.715	2.83E−04	cell division cycle 42	rno-miR-137-3p
Downregulated					
Zc2hc1b	100,361,672	− 5.757	5.60E−05	zinc finger	
Zc3hav1	252,832	− 5.510	5.60E−05	zinc finger CCCH type	
Ptms	83,801	− 4.221	5.60E−05	parathymosin	
Hmx1	360,960	− 3.841	6.64E−05	H6 family homeobox 1	
Etv3	295,297	− 5.703	7.57E−05	ets variant 3	
Kmt2d	100,362,634	− 3.476	7.57E−05	lysine (K)-specific methyltransferase 2D	
Gimap8	500,112	− 5.896	7.97E−05	GTPase	
Sfpq	252,855	− 3.246	9.59E−05	splicing factor proline/glutamine-rich	
Cass4	296,409	− 3.950	9.66E−05	Cas scaffolding protein family member 4	
Elac1	307,604	− 3.324	9.66E−05	elaC ribonuclease Z 1	
Pamr1	311,252	− 3.305	9.66E−05	peptidase domain containing associated with muscle regeneration 1	
Kcnj9	116,560	− 3.897	1.01E−04	potassium channel	
Rmrp	29,536	− 3.885	1.25E−04	RNA component of mitochondrial RNA processing endoribonuclease	
Ift57	303,968	− 5.138	1.25E−04	intraflagellar transport 57	
Hes7	287,423	− 4.184	1.27E−04	hes family bHLH transcription factor 7	
Ubxn1	293,719	− 3.527	1.54E−04	UBX domain protein 1	
Araf	64,363	− 3.510	1.73E−04	A-Raf proto-oncogene	
Foxe1	192,274	− 5.828	1.76E−04	forkhead box E1	
Lzts3	280,670	− 3.317	1.77E−04	leucine zipper	
Ccnf	117,524	− 3.384	1.78E−04	cyclin F	
Hs3st3b1	303,218	− 4.901	1.85E−04	heparan sulfate (glucosamine) 3-O-sulfotransferase 3B1	
Aig1	292,486	− 3.523	1.85E−04	androgen-induced 1	
Bcl11a	305,589	− 4.236	1.92E−04	B-cell CLL/lymphoma 11A (zinc finger protein)	
Tbx21	303,496	− 3.445	2.03E−04	T-box 21	
Avil	79,253	− 3.577	2.11E−04	advillin	
Plcb2	85,240	− 3.427	2.17E−04	phospholipase C	
RatNP-3b	498,659	− 4.205	2.21E−04	defensin RatNP-3 precursor	
Srcap	361,652	− 3.261	2.23E−04	Snf2-related CREBBP activator protein	
Lmnb1	116,685	− 3.286	2.24E−04	lamin B1	
Elf2	361,944	− 4.757	2.25E−04	E74-like factor 2	rno-miR-29b-3p
Phtf1	252,962	− 5.586	2.34E−04	putative homeodomain transcription factor 1	
Mospd2	363,463	− 3.669	2.39E−04	motile sperm domain containing 2	rno-miR-29b-3p

(a) List of most significant up and downregulated DEGs following perinatal alcohol exposure compared to saline control. (b) List of most significant up and downregulated DEGs following perinatal nicotine-alcohol exposure compared to saline control. (c) List of most significant up and downregulated DEGs following perinatal nicotine-alcohol exposure compared to alcohol exposure. The genes were listed after Benjamini–Hochberg corrections with adjusted p value < 0.001, q value < 0.05 and absolute log2 fold change > 1. Predicted miRNA targets were also included in both lists.

### miRNAs and their target gene profiling

**Figure 2:**
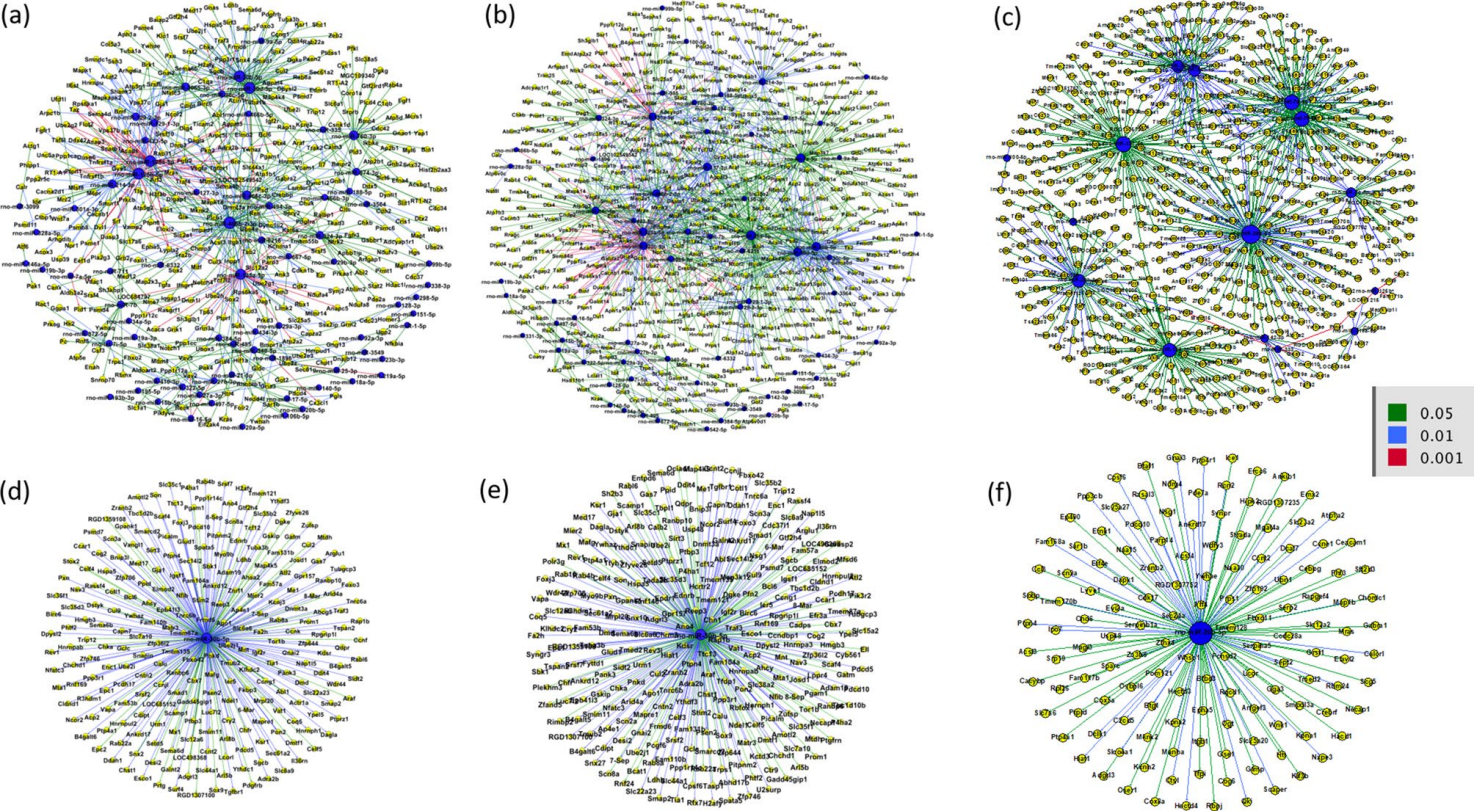
Integrated analysis of the predicted and validated miRNA-mRNA target network. Following perinatal (**a**) alcohol exposure compared to saline control, (**b**) nicotine–alcohol exposure compared to saline control, and (**c**) nicotine–alcohol exposure compared to alcohol exposure. For both (**d**), following alcohol exposure compared to saline control and (**e**), following nicotine–alcohol exposure compared to saline control, using negative correlation, rno-miR-30b-5p was predicted to target the greatest number of genes within our DEGs. For (**f**) following nicotine–alcohol exposure compared to alcohol exposure, using negative correlation, rno-miR-26b-5p was predicted to target the greatest number of genes within our DEGs.

Using the list of our DEGs and DEmiRs following each treatment group, predicted and validated miRNA and mRNA targets were found and plotted using MultiMiR package. After alcohol treatment, 770 miRNA–gene target pairs with 455 nodes and 1,148 miRNA–gene target pairs with 581 nodes following nicotine–alcohol treatment were found based on conserved prediction sites. Figure 2 shows the predicted networks for miRNAs and their target genes after (a) perinatal alcohol compared to the saline control group, (b) perinatal nicotine–alcohol treatment compared to the saline control group, and (c) perinatal nicotine–alcohol treatment compared to the alcohol group. Among the DEmiRs, mir-30b was found to have the greatest number of connections to DEGs in the perinatal alcohol vs. saline comparison as well as the perinatal nicotine–alcohol vs. saline comparison. Figure 2d,e illustrate the miR-30b DEGs after alcohol and nicotine–alcohol exposure, respectively. miR-30b was predicted to target Gnai2 and Cotl1 following alcohol exposure and Gnai2 and Bnip3l following nicotine–alcohol exposure (Table 2). Among the DEmiRs, mir-26b was found to have the greatest number of connections to DEGs in the perinatal nicotine–alcohol group when compared against the alcohol treatment group as shown in Fig. 2f. miR-26b was predicted to target Nxpe3 following nicotine–alcohol exposure vs. alcohol exposure (Table 2).

### Enriched pathway analysis, and target predictions

**Figure 3:**
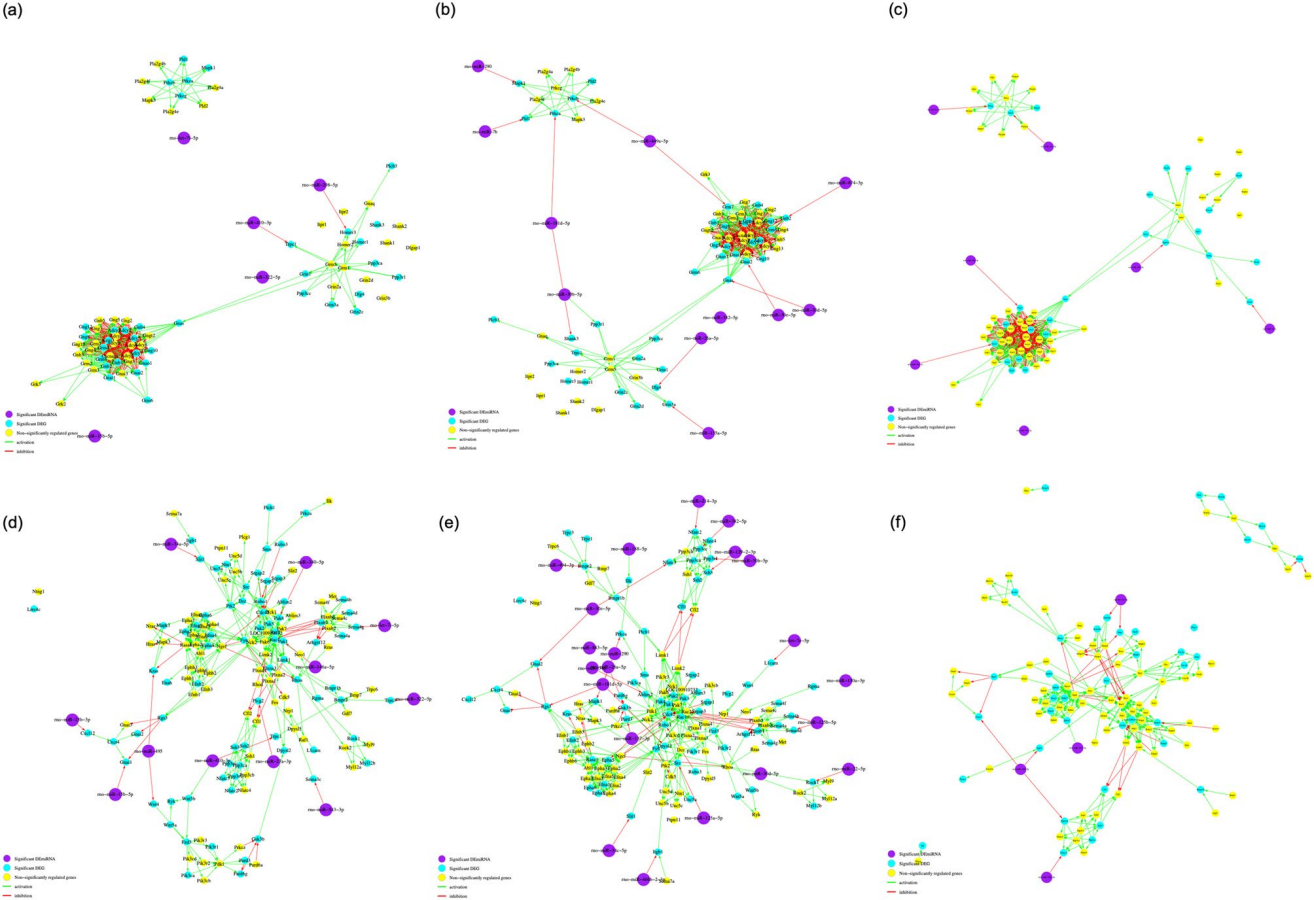
Enriched KEGG pathways were shown. Glutamatergic synapse pathway was shown in (**a**–**c**) following perinatal alcohol compared to saline control, nicotine–alcohol compared to saline control, and nicotine–alcohol compared to alcohol exposure in DA neurons, respectively, (p < 0.001 for alcohol compared to saline control, p < 0.01 for nicotine–alcohol compared to saline control, and p > 0.05 for nicotine–alcohol compared to alcohol exposure). Axon guidance pathway was shown in (**d**–**f**) following perinatal alcohol compared to saline control, nicotine–alcohol compared to saline control, and nicotine–alcohol compared to alcohol exposure in DA neurons, (p < 0.01 for alcohol compared to saline, p < 0.01 for nicotine–alcohol compared to saline, and p > 0.05 for nicotine–alcohol compared to alcohol exposure).

The multiMiR^51^ R package was used to find the miRNA and mRNA validated and predicted target pairings. This was done using inversely regulated DEmiRs and DEGs. Using DAVID v6.8^52,53^, we further analyzed our DEG and DEmiR lists and looked across the significantly enriched Kyoto Encyclopedia of Genes and Genomes (KEGG)^54–56^ pathways following each treatment to better understand the systemic effect of perinatal alcohol or nicotine–alcohol exposure not only on the neuronal level, but also throughout other biological pathways in the body. The results summarized in Table 3, revealed many enriched KEGG pathways associated with cancer (p < 0.001 for alcohol and p < 0.01 for nicotine–alcohol), Glutamatergic synapse (p < 0.001 for alcohol and p < 0.01 for nicotine–alcohol), axon guidance (p < 0.01 for alcohol and p < 0.01 for nicotine–alcohol), and Mitogen-Activated Protein Kinase (MAPK) signaling pathway (p < 0.001 for alcohol and p < 0.001 for nicotine–alcohol). The axonal guidance KEGG pathway was significantly enriched among the upregulated genes following both treatment groups. Additionally, the glutamatergic synapse KEGG pathway was enriched among the significantly differentially expressed up and downregulated genes following both treatment groups. This information was then used to obtain further insight regarding the enriched pathways, including possible underlying biological processes, and to identify potential regulatory points across treatment groups. KEGGgraph^57^ was additionally used to generate gene networks. When perinatal nicotine–alcohol treatment group was compared to the alcohol treatment group as shown in Supplementary Table S1, pathways in cancer, long term potentiation, Huntington’s disease and Parkinson’s disease were among the many biological pathways that were significantly altered. Figure 3a–c show the miRNA–gene network from the Glutamatergic synapse after perinatal alcohol vs. saline, perinatal nicotine–alcohol vs. saline, and perinatal nicotine–alcohol vs. alcohol treatments, respectively (p < 0.001 for alcohol, p < 0.01 for nicotine–alcohol, and p > 0.05 for nicotine–alcohol vs. alcohol). Figure 3d–f illustrate the axon guidance KEGG pathways after perinatal alcohol vs. saline, perinatal nicotine–alcohol vs. saline, and perinatal nicotine–alcohol vs. alcohol treatments respectively (p < 0.01 for alcohol, p < 0.01 for nicotine–alcohol, and p > 0.05 for nicotine–alcohol vs. alcohol).

**Table 3:** KEGG pathways enriched by up and downregulated DEGs and the corresponding genes identified in pathway analysis (a) following perinatal alcohol exposure (b) following perinatal nicotine and alcohol exposure.

Perinatal alcohol exposure			Perinatal nicotine-alcohol exposure		
KEGG term	P value	Genes	KEGG term	P value	Genes
**Downregulated**			**Downregulated**		
Glutamatergic synapse	8.53E−04	TRPC1, GRM2, HOMER3, GNG13, PRKCG, GRIA3, SLC38A1, GRIA4, SLC1A1	MicroRNAs in cancer	1.34E−02	RECK, BAK1, NOTCH1, KRAS, PAK4, MDM4, PDCD4
Protein processing in endoplasmic reticulum	8.90E−03	ATF6, UBE2E3, HERPUD1, UFD1L, RNF5, FBXO2, DNAJB12, SAR1B, EIF2AK4	Metabolic pathways	2.24E−02	COASY, PPCS, HPRT1, EXTL1, ALDH3A2, HIBADH, GLDC, GOT2, CRYL1, AGPAT5, ST3GAL5, ST3GAL6, GGPS1, GALNT18, ATP6V0D1, IPMK, ALDOART2, SPTLC1, NME7, HYI, PGLS, PANK2, HSD11B1, LCLAT1, GPAM, SMPD3, UGP2
MicroRNAs in cancer	1.23E−02	RECK, DNMT3A, NOTCH1, KRAS, PAK4, PRKCG, MDM4, PDCD4	Pantothenate and CoA biosynthesis	2.72E−02	COASY, PANK2, PPCS
cAMP signaling pathway	2.04E−02	VAV3, RAC1, PPP1R12A, HTR4, NFKBIA, GRIA3, PAK1, GRIA4, VAV2	Biosynthesis of antibiotics	3.02E−02	GOT2, ALDOART2, PGLS, GGPS1, UGP2, NME7, ALDH3A2, GLDC
Natural killer cell mediated cytotoxicity	2.79E−02	KRAS, VAV3, RAC1, PRKCG, PAK1, VAV2	Signaling pathways regulating pluripotency of stem cells	4.26E−02	ACVR2A, WNT1, KRAS, SOX2, ID4, AXIN2
Fc epsilon RI signaling pathway	3.19E−02	KRAS, VAV3, RAC1, PRKCG, VAV2	Glycerolipid metabolism	4.66E−02	AGPAT5, LCLAT1, GPAM, ALDH3A2
B cell receptor signaling pathway	3.19E−02	KRAS, VAV3, RAC1, NFKBIA, VAV2	T cell receptor signaling pathway	5.54E−02	KRAS, PAK4, NFKBIA, PAK1, VAV2
T cell receptor signaling pathway	3.60E−02	KRAS, VAV3, PAK4, NFKBIA, PAK1, VAV2	Glutamatergic synapse	7.24E−02	TRPC1, GRM2, HOMER3, GRIA3, GRIA4
Biosynthesis of antibiotics	3.72E−02	GOT2, ALDOART2, PGLS, HK2, GGPS1, UGP2, NME7, ALDH3A2, GLDC	Protein processing in endoplasmic reticulum	8.00E−02	ATF6, UBE2E3, BAK1, HERPUD1, FBXO2, DNAJB12
Carbon metabolism	5.98E−02	GOT2, ALDOART2, PGLS, HK2, GLDC, PC	Hypertrophic cardiomyopathy (HCM)	9.62E−02	ACTC1, CACNG8, ITGB4, CACNB2
Fc gamma R-mediated phagocytosis	6.06E−02	VAV3, RAC1, PRKCG, PAK1, VAV2	**Upregulated**		
Regulation of actin cytoskeleton	8.43E−02	KRAS, VAV3, PAK4, PIKFYVE, RAC1, PPP1R12A, PAK1, VAV2	Hippo signaling pathway	7.74E−05	PARD3, YWHAZ, MOB1A, APC2, SOX2, BMPR2, TCF7L2, ACTG1, FRMD6, DLG4, LIMD1, YAP1, FGF1, BMP4, TGFBR1, SMAD1, TEAD3, PPP1CC, YWHAE, DVL1, PPP1CA, CCND1, YWHAH, YWHAQ, WNT7A, BMPR1A
			MAPK signaling pathway	1.56E−04	FGFR2, FGFR1, FGFR3, GRB2, MRAS, MKNK2, PPP3R1, CACNB1, FASLG, SRF, CDC42, TNFRSF1A, MAPT, SOS2, FGF1, NFATC3, CHUK, CACNA2D1, NLK, MAP2K3, TGFBR1, TAB2, PRKCB, RPS6KA5, MAP4K3, MAPK1, MAP4K4, DUSP3, RPS6KA1, RASGRF1, MAPK14, NTRK2, PDGFRA, RAP1B, CACNA1B, DUSP6
Chemokine signaling pathway	8.84E−02	KRAS, VAV3, RAC1, GNG13, NFKBIA, PAK1, VAV2	Adherens junction	1.64E−04	PTPN6, FGFR1, PARD3, PTPRF, BAIAP2, TGFBR1, NLK, CREBBP, TCF7L2, IQGAP1, SRC, ACTG1, MAPK1, CDC42, PTPN1, SSX2IP
Ubiquitin mediated proteolysis	9.72E−02	UBE2E3, UBE2Z, FBXO2, RHOBTB1, CDC23, NEDD4L	Renal cell carcinoma	2.64E−04	GRB2, CREBBP, RBX1, CDC42, CUL2, MAPK1, HIF1A, ETS1, SOS2, SLC2A1, ARAF, VEGFA, TGFA, RAP1B, PIK3R2
**Upregulated**			GABAergic synapse	3.86E-04	SLC38A5, GABRA2, GABRA1, GNAO1, GNAI2, SLC6A1, GABRB2, ADCY6, GABBR1, SRC, PRKCB, GNB2, TRAK2, GNB1, GLS, GNG5, CACNA1B
Endocytosis	1.96E−06	ARFGAP1, FGFR2, PARD3, FGFR3, CHMP5, CAPZA2, SNX2, VPS37B, VPS37C, SNX4, CLTC, SRC, CDC42, SMAP2, AP2B1, FOLR2, SH3GLB1, DNM3, RAB8A, PLD1, TGFBR1, RAB4A, RT1-A2, RT1-A1, ARPC1B, RAB31, PSD, ACAP3, IST1, ARPC5L, ARF3, IGF2R, RAB22A, PDGFRA, SH3KBP1, HGS, SNX32, SMURF1, BIN1, RT1-N2	Endocytosis	4.06E−04	ARFGAP1, FGFR2, PARD3, FGFR3, CHMP5, CAPZA2, VPS37B, VPS37C, CYTH3, CLTC, SRC, CDC42, SMAP2, FOLR2, SH3GLB1, DNM3, RAB8A, PLD1, TGFBR1, RAB4A, RT1-A2, RT1-A1, ARPC1B, RAB31, CHMP1A, PSD, ACAP3, IST1, ARF3, IGF2R, RAB22A, PDGFRA, SH3KBP1, HGS, SMURF1, BIN1
MicroRNAs in cancer	4.80E−05	DNMT3A, FGFR3, GRB2, CREBBP, BMPR2, TP63, ZEB2, UBE2I, CCNG1, PRKCB, DDIT4, RPS6KA5, SPRY2, EZR, CDKN1B, GLS, SOS2, NOTCH4, VEGFA, PDGFRA, PDGFRB, SHC1, ABL1, BMF	MicroRNAs in cancer	4.92E−04	DNMT3A, FGFR3, APC2, GRB2, CREBBP, BMPR2, TP63, ZEB2, UBE2I, CCNG1, PRKCB, DDIT4, RPS6KA5, CCND1, EZR, CDKN1B, GLS, SOS2, VEGFA, PDGFRA, SHC1, ABL1, BMF
MAPK signaling pathway	5.85E−05	FGFR2, FGFR1, FGFR3, GRB2, MRAS, MKNK2, PPP3R1, CACNB1, MAPKAPK2, SRF, CDC42, TNFRSF1A, MAPT, SOS2, FGF1, NFATC3, CHUK, CACNA2D1, LAMTOR3, MAP2K3, TGFBR1, TAB2, PRKCB, RPS6KA5, MAPK1, MAP4K4, DUSP3, RPS6KA1, RASGRF1, MAPK14, NTRK2, PDGFRA, PDGFRB, RAP1B, DUSP6	Pathways in cancer	1.21E−03	FGFR2, FGFR1, FGFR3, APC2, GNAI2, GRB2, ADCY6, MITF, FOXO1, TFG, FASLG, ITGB1, TCF7L2, SUFU, TPM3, RBX1, EDNRB, CUL2, CDC42, SLC2A1, SOS2, TGFA, FGF1, GNG5, CHUK, TRAF3, PIK3R2, BMP4, CTBP1, RALBP1, TGFBR1, CREBBP, DVL1, PRKCB, MAPK1, CCND1, HIF1A, CDKN1B, GNB2, GNB1, VEGFA, ARAF, PDGFRA, GNAS, ABL1, WNT7A
Adherens junction	2.26E−04	PTPN6, FGFR1, PARD3, PTPRF, BAIAP2, TGFBR1, CREBBP, TCF7L2, IQGAP1, SRC, ACTG1, MAPK1, CDC42, PTPN1, SSX2IP	Neurotrophin signaling Pathway	1.49E−03	GRB2, FASLG, FOXO3, KIDINS220, YWHAE, RPS6KA5, CDC42, MAPK1, RPS6KA1, MAPK14, SOS2, NTRK2, PSEN2, SH2B3, SHC1, RAP1B, ABL1, ARHGDIA, ARHGDIB, PIK3R2
Pathways in cancer	3.30E−04	FGFR2, FGFR1, FGFR3, GNAI2, GRB2, MITF, FOXO1, TFG, HDAC1L, ITGB1, TCF7L2, SUFU, TPM3, RBX1, EDNRB, CUL2, CDC42, SLC2A1, SOS2, TGFA, PLCB1, FGF1, TRAF4, CHUK, TRAF3, BMP4, CTBP1, RALBP1, TGFBR1, CREBBP, CDK2, DVL1, PRKCB, MAPK1, HIF1A, CDKN1B, GNB2, GNB1, VEGFA, ARAF, PDGFRA, PDGFRB, GNAS, ABL1, WNT7A	Central carbon metabolism in cancer	1.70E−03	FGFR2, FGFR1, FGFR3, PFKL, PGAM1, SIRT6, SIRT3, MAPK1, HIF1A, GLS, SLC2A1, PDGFRA, PIK3R2
Hippo signaling pathway	3.70E−04	BMP4, YWHAZ, PARD3, TGFBR1, SOX2, BMPR2, TEAD3, SMAD1, PPP1CC, TCF7L2, YWHAE, DVL1, ACTG1, PPP1CA, YWHAH, FRMD6, CSNK1D, YWHAQ, DLG4, YAP1, FGF1, WNT7A, BMPR1A	Proteoglycans in cancer	2.26E−03	FGFR1, GRB2, MRAS, PPP1R12C, FASLG, ITGB1, IQGAP1, SRC, PXN, ACTG1, CDC42, EZR, ANK1, SOS2, PIK3R2, PTPN6, PPP1CC, DDX5, PRKCB, MAPK1, PPP1CA, CCND1, HIF1A, MAPK14, VEGFA, ARAF, WNT7A
Renal cell carcinoma	3.88E−04	GRB2, CREBBP, RBX1, CDC42, CUL2, MAPK1, HIF1A, ETS1, SOS2, SLC2A1, ARAF, VEGFA, TGFA, RAP1B	Retrograde endocannabinoid signaling	2.52E−03	GABRA2, GABRA1, GNAO1, GNAI2, GABRB2, ADCY6, GRIA4, PRKCB, MAPK1, DAGLA, SLC17A6, GNB2, GNB1, MAPK14, MGLL, GNG5, CACNA1B
Glutamatergic synapse	4.42E−04	DLGAP1, PLD1, GNAO1, GNAI2, GRIK1, PPP3R1, GRIN3A, GRIA4, KCNJ3, SHANK3, PRKCB, MAPK1, SLC17A6, GNB2, GNB1, GLS, DLG4, GNAS, PLCB1	Glycerophospholipid metabolism	2.89E−03	PLD3, CHKA, CRLS1, CDIPT, PLD1, PLA2G15, CHKB, TAZ, LYPLA2, CHPT1, DGKE, DGKG, PLA2G3, AGPAT4, PTDSS1, AGPAT3
Gap junction	5.20E−04	GNAI2, GRB2, TUBA3B, GJA1, SRC, PRKCB, MAPK1, CSNK1D, SOS2, TUBB5, PDGFRA, PDGFRB, GNAS, PLCB1, TUBA1A, TUBA1B	Glutamatergic synapse	3.21E−03	PLD1, GNAO1, GNAI2, GRIK1, ADCY6, PPP3R1, GRIN3A, GRIA4, SHANK3, PRKCB, MAPK1, SLC17A6, GNB2, GNB1, GLS, DLG4, GNAS, GNG5
Central carbon metabolism in cancer	7.74E−04	FGFR2, FGFR1, FGFR3, PFKL, PGAM1, SIRT6, SIRT3, MAPK1, HIF1A, GLS, SLC2A1, PDGFRA, PDGFRB	Regulation of actin cytoskeleton	6.40E−03	FGFR2, FGFR1, FGFR3, APC2, MRAS, SSH3, PPP1R12C, ABI2, ITGB1, IQGAP1, SRC, PXN, ACTG1, CDC42, EZR, SOS2, BRK1, FGF1, PIK3R2, LIMK2, BAIAP2, PPP1CC, MAPK1, ARPC1B, PPP1CA, ARAF, PDGFRA
Regulation of actin cytoskeleton	8.87E−04	FGFR2, FGFR1, ENAH, FGFR3, SSH3, MRAS, PPP1R12C, ABI2, ITGB1, PXN, IQGAP1, SRC, ACTG1, CDC42, EZR, SOS2, BRK1, FGF1, LIMK2, BAIAP2, PPP1CC, MAPK1, ARPC1B, PPP1CA, ARPC5L, ARAF, PDGFRA, PDGFRB	Spliceosome	8.06E−03	SRSF10, TRA2B, DDX5, RBMX, HNRNPU, SF3A3, SMNDC1, HNRNPA3, HNRNPM, SRSF2, SRSF5, SRSF4, SRSF7, SRSF6, USP39, SNRNP70, HNRNPC, ACIN1, DDX42
Glycerophospholipid metabolism	3.32E−03	PLD3, CHKA, CRLS1, CDIPT, PLD1, CHKB, TAZ, LYPLA2, CHPT1, DGKE, DGKG, PLA2G3, AGPAT4, PTDSS1, AGPAT3	Axon guidance	9.55E−03	ABLIM2, GNAI2, LIMK2, PPP3R1, L1CAM, DPYSL2, ITGB1, SLIT1, EPHA5, MAPK1, CDC42, SEMA6B, SEMA6D, UNC5A, SEMA4B, SEMA4D, ABL1, NFATC3
Neurotrophin signaling pathway	3.33E−03	GRB2, MAPKAPK2, FOXO3, YWHAE, RPS6KA5, CDC42, MAPK1, RPS6KA1, MAPK14, SOS2, NTRK2, PSEN2, CALM3, SHC1, RAP1B, ABL1, ARHGDIA, ARHGDIB	Ubiquitin mediated proteolysis	1.08E−02	SYVN1, UBE4A, ANAPC5, UBE2G1, UBE2G2, UBE2J1, BIRC6, UBE2I, CDC34, UBE2H, UBOX5, RBX1, CUL3, CUL2, PIAS4, UBE2K, KLHL9, SMURF1, TRIP12
Rap1 signaling pathway	3.68E−03	FGFR2, FGFR1, PARD3, FGFR3, GNAI2, MRAS, ITGB1, APBB1IP, SRC, ACTG1, CDC42, PLCB1, FGF1, GNAO1, MAP2K3, PRKCB, MAPK1, MAPK14, VEGFA, PDGFRA, CALM3, PDGFRB, GNAS, RAP1B, EFNA4, PRKD3	Oxytocin signaling pathway	1.20E−02	MYL6, CACNA2D1, CAMK1G, GNAO1, GNAI2, ADCY6, PPP1R12C, CACNB1, PPP3R1, NPR1, PPP1CC, SRC, PRKCB, ACTG1, MAPK1, PPP1CA, CCND1, PRKAA1, GNAS, NFATC3
Axon guidance	3.93E−03	ABLIM2, GNAI2, LIMK2, PPP3R1, DPYSL2, ITGB1, SLIT1, EPHA5, MAPK1, CDC42, SEMA6B, SEMA6D, UNC5A, SEMA4B, EFNA4, SEMA4D, ABL1, NFATC3	Morphine addiction	1.35E−02	GABRA2, GABRA1, GNAO1, GNAI2, GABRB2, ADCY6, GABBR1, PRKCB, GNB2, PDE2A, GNB1, GNAS, GNG5, CACNA1B
Ras signaling pathway	4.51E−03	FGFR2, FGFR1, FGFR3, GRB2, MRAS, CDC42, SOS2, SHC1, FGF1, CHUK, PLD1, RALBP1, PRKCB, MAPK1, GNB2, ETS1, GNB1, RASGRF1, VEGFA, PDGFRA, PDGFRB, CALM3, RAP1B, EFNA4, ABL1, KSR1, PLA2G3	Ras signaling pathway	1.40E−02	FGFR2, FGFR1, FGFR3, GRB2, MRAS, FASLG, CDC42, SOS2, SHC1, FGF1, GNG5, CHUK, PIK3R2, PLD1, RALBP1, PRKCB, MAPK1, GNB2, ETS1, GNB1, RASGRF1, VEGFA, PDGFRA, RAP1B, PLA2G3, ABL1, KSR1
Oxytocin signaling pathway	4.69E−03	MYL6, CACNA2D1, GNAO1, GNAI2, PPP1R12C, CACNB1, PPP3R1, NPR1, PPP1CC, KCNJ3, SRC, PRKCB, ACTG1, MAPK1, PPP1CA, CALM3, PRKAA1, GNAS, PLCB1, NFATC3	Alcoholism	1.41E−02	HIST2H2AA3, GNAO1, GNAI2, GRB2, LOC684797, HDAC10, GRIN3A, PPP1CC, HIST2H4, MAPK1, PPP1CA, GNB2, GNB1, HIST1H4B, SOS2, ARAF, NTRK2, H2AFY, SHC1, H3F3B, GNAS, GNG5
Proteoglycans in cancer	6.01E−03	PTPN6, FGFR1, GRB2, MRAS, PPP1R12C, PPP1CC, DDX5, ITGB1, PXN, SRC, IQGAP1, PRKCB, ACTG1, CDC42, MAPK1, PPP1CA, EZR, HIF1A, ANK3, MAPK14, SOS2, ARAF, VEGFA, WNT7A	Protein processing in endoplasmic reticulum	1.67E−02	SYVN1, DERL1, UBE2G1, ERP29, UBE2G2, UBE2J1, MAN1B1, CALR, SEC63, SSR1, RBX1, HYOU1, ATXN3, STT3A, HSPA5, SEC24C, UGGT1, SSR2, DNAJC1, SEC61G, SEC61A2
Notch signaling pathway	6.01E−03	DTX4, CTBP1, APH1A, DTX2, PSEN2, NOTCH4, CREBBP, HDAC1L, NCOR2, DVL1	FoxO signaling pathway	1.69E−02	GRB2, NLK, TGFBR1, CREBBP, FASLG, FOXO1, FOXO3, MAPK1, PRMT1, CCND1, CDKN1B, MAPK14, SOS2, ARAF, PRKAA1, BCL6, CHUK, PIK3R2
Long-term potentiation	9.06E−03	MAPK1, PPP1CA, RPS6KA1, ARAF, CREBBP, PPP3R1, CALM3, RAP1B, PPP1CC, PLCB1, PRKCB	Chronic myeloid leukemia	1.71E−02	MAPK1, CTBP1, CCND1, CDKN1B, GRB2, TGFBR1, SOS2, ARAF, SHC1, ABL1, CHUK, PIK3R2
cGMP-PKG signaling pathway	9.26E−03	KCNMA1, ATP1B1, GNAI2, SLC25A5, PPP3R1, NPR1, PPP1CC, SRF, VDAC1, ATP2B1, EDNRB, MAPK1, PPP1CA, ATP2A2, PDE2A, GTF2IRD1, CALM3, ADRA2B, PLCB1, NFATC3	Phosphatidylinositol signaling system	1.88E−02	INPP1, CDIPT, IMPAD1, PIK3C2A, TMEM55B, PRKCB, MTMR2, MTMR3, MTMR14, DGKE, DGKG, SYNJ2, PLCD4, PIK3R2
Choline metabolism in cancer	1.41E−02	CHKA, PLD1, SLC44A1, GRB2, CHKB, CHPT1, PRKCB, MAPK1, HIF1A, DGKE, DGKG, SOS2, PDGFRA, PDGFRB	Rap1 signaling pathway	2.05E−02	FGFR2, FGFR1, TLN1, PARD3, FGFR3, GNAI2, MRAS, ADCY6, ITGB1, APBB1IP, SRC, ACTG1, CDC42, RAPGEF6, FGF1, PIK3R2, GNAO1, MAP2K3, PRKCB, MAPK1, MAPK14, VEGFA, PDGFRA, GNAS, RAP1B
Spliceosome	1.57E−02	SRSF10, TRA2B, WBP11, DDX5, RBMX, HNRNPU, SMNDC1, HNRNPM, SRSF2, SRSF5, SRSF4, SRSF7, SRSF6, USP39, ACIN1, SNRNP70, DDX42	Signaling pathways regulating pluripotency of stem cells	2.19E−02	BMP4, FGFR2, SMARCAD1, FGFR1, FGFR3, APC2, GRB2, SOX2, BMPR2, SMAD1, DVL1, MAPK1, MAPK14, PCGF6, WNT7A, PIK3R2, KAT6A, BMPR1A
Retrograde endocannabinoid signaling	1.64E−02	GABRA2, GNAO1, GNAI2, GRIA4, KCNJ3, PRKCB, MAPK1, DAGLA, SLC17A6, GNB2, GNB1, MAPK14, MGLL, PLCB1	cGMP-PKG signaling pathway	2.23E−02	ATP1B1, GNAI2, SLC25A4, SLC25A5, ADCY6, PPP3R1, NPR1, PPP1CC, SRF, VDAC1, ATP2B1, EDNRB, MAPK1, PPP1CA, ATP2A2, PDE2A, GTF2I, GTF2IRD1, ADRA2B, NFATC3
Alcoholism	2.10E−02	HIST2H2AA3, GNAO1, GNAI2, GRB2, LOC684797, HDAC1L, GRIN3A, PPP1CC, MAPK1, PPP1CA, GNB2, GNB1, SOS2, ARAF, NTRK2, H2AFY, CALM3, SHC1, H3F3B, GNAS	Gap junction	2.23E−02	GNAI2, GRB2, TUBA3B, ADCY6, GJA1, SRC, PRKCB, MAPK1, SOS2, PDGFRA, GNAS, TUBA1A, TUBA1B
Phosphatidylinositol signaling system	2.21E−02	INPP1, MTMR3, MTMR14, IMPAD1, CDIPT, DGKE, DGKG, CALM3, PLCD4, SYNJ2, PLCB1, TMEM55B, PRKCB	Choline metabolism in cancer	2.75E−02	CHKA, PLD1, SLC44A1, GRB2, CHKB, CHPT1, PRKCB, MAPK1, HIF1A, DGKE, DGKG, SOS2, PDGFRA, PIK3R2
PI3K-Akt signaling pathway	2.22E−02	PHLPP1, CSF3, FGFR2, FGFR1, YWHAZ, FGFR3, GRB2, PPP2R5C, FOXO3, ITGB1, CDC37, SOS2, GYS1, PRKAA1, MLST8, FGF1, CHUK, IL7, PKN2, COL5A3, YWHAE, CDK2, DDIT4, MAPK1, YWHAH, CDKN1B, GNB2, GNB1, VEGFA, YWHAQ, PDGFRA, PDGFRB, EFNA4	Notch signaling pathway	3.03E−02	DTX4, CTBP1, APH1A, DTX2, PSEN2, CREBBP, RBPJ, NCOR2, DVL1
Chronic myeloid leukemia	2.35E−02	MAPK1, CTBP1, CDKN1B, GRB2, TGFBR1, SOS2, ARAF, SHC1, HDAC1L, ABL1, CHUK	Endometrial cancer	3.03E−02	MAPK1, CCND1, APC2, GRB2, SOS2, ARAF, FOXO3, TCF7L2, PIK3R2
Circadian entrainment	2.38E−02	GNAO1, GNAI2, ADCYAP1R1, GRIA4, KCNJ3, PRKCB, RPS6KA5, MAPK1, GNB2, GNB1, CALM3, GNAS, PLCB1	Protein export	3.47E−02	MGC109340, HSPA5, SRPRB, SEC63, SEC61G, SEC61A2
Glioma	2.47E−02	MAPK1, GRB2, SOS2, ARAF, PDGFRA, CALM3, TGFA, PDGFRB, SHC1, PRKCB	Lysosome	3.73E−02	PLA2G15, AP4E1, ACP2, CD164, CLTC, M6PR, DNASE2, SLC11A2, SLC17A5, GNPTAB, IGF2R, CTSD, CTSB, GGA1, GGA3, IDUA
GABAergic synapse	2.58E−02	SLC38A5, GABRA2, GNAO1, GNB2, GNAI2, TRAK2, SLC6A1, GNB1, GLS, GABBR1, SRC, PRKCB	Pancreatic cancer	3.75E−02	MAPK1, CDC42, CCND1, RALBP1, TGFBR1, VEGFA, ARAF, TGFA, CHUK, PIK3R2
Melanogenesis	2.75E−02	MAPK1, EDNRB, GNAO1, GNAI2, CREBBP, MITF, CALM3, GNAS, PLCB1, TCF7L2, WNT7A, DVL1, PRKCB	Basal transcription factors	3.93E−02	TAF5L, GTF2I, GTF2IRD1, TAF8, TAF9B, GTF2H4, ERCC3, ERCC2
Glucagon signaling pathway	2.94E−02	LDHB, CREBBP, PGAM1, PPP3R1, ACACA, FOXO1, PRMT1, SLC2A1, GYS1, CALM3, PRKAA1, GNAS, PLCB1	Hepatitis B	3.96E−02	YWHAZ, GRB2, TGFBR1, CREBBP, FASLG, SRC, STAT2, PRKCB, IKBKE, MAPK1, CCND1, CDKN1B, YWHAQ, TICAM2, NFATC3, CHUK, PIK3R2
GnRH signaling pathway	3.70E−02	MAPK1, CDC42, PLD1, GRB2, MAP2K3, MAPK14, SOS2, CALM3, GNAS, PLCB1, SRC, PRKCB	Glioma	4.09E−02	MAPK1, CCND1, GRB2, SOS2, ARAF, PDGFRA, TGFA, SHC1, PRKCB, PIK3R2
Ubiquitin mediated proteolysis	3.98E−02	UBE4A, ANAPC5, UBE2G1, UBE2G2, UBE2J1, BIRC6, UBE2I, CDC34, UBE2H, UBOX5, RBX1, CUL3, CUL2, UBE2K, SMURF1, TRIP12	Non-small cell lung cancer	4.46E−02	MAPK1, CCND1, GRB2, SOS2, ARAF, TGFA, FOXO3, PRKCB, PIK3R2
VEGF signaling pathway	4.04E−02	MAPK1, CDC42, MAPK14, VEGFA, PPP3R1, MAPKAPK2, PXN, SRC, PRKCB	Mucin type O-Glycan biosynthesis	4.61E−02	GALNT2, GALNT1, C1GALT1C1, GALNT7, GALNT14, B4GALT5
Phagosome	4.78E−02	DYNC1LI2, TUBA3B, MRC2, CALR, ITGB1, CANX, M6PR, ACTG1, RT1-A2, RT1-A1, CORO1A, TUBB5, HGS, SCARB1, TUBA1A, TUBA1B, DYNC1I2, SEC61G, SEC61A2, RT1-N2	Platelet activation	5.85E−02	TLN1, GNAI2, ADCY6, COL5A3, PPP1CC, ITGB1, APBB1IP, SRC, ACTG1, MAPK1, PPP1CA, GP1BB, MAPK14, RAP1B, GNAS, PIK3R2
FoxO signaling pathway	6.05E−02	GRB2, TGFBR1, CREBBP, FOXO1, FOXO3, CDK2, MAPK1, PRMT1, CDKN1B, MAPK14, SOS2, ARAF, PRKAA1, BCL6, CHUK	VEGF signaling pathway	6.28E−02	MAPK1, CDC42, MAPK14, VEGFA, PPP3R1, PXN, SRC, PRKCB, PIK3R2
Adrenergic signaling in cardiomyocytes	6.67E−02	ATP1B1, CACNA2D1, GNAI2, PPP2R5C, CACNB1, PPP1CC, TPM3, RPS6KA5, ATP2B1, MAPK1, PPP1CA, MAPK14, CALM3, GNAS, PLCB1	Nicotine addiction	6.31E−02	GABRA2, GABRA1, SLC17A6, GABRB2, GRIA4, GRIN3A, CACNA1B
Hepatitis B	7.00E−02	YWHAZ, GRB2, TGFBR1, CREBBP, CDK2, SRC, PRKCB, STAT2, IKBKE, MAPK1, CDKN1B, YWHAQ, TICAM2, NFATC3, CHUK	Bladder cancer	6.31E−02	RPS6KA5, MAPK1, CCND1, FGFR3, VEGFA, ARAF, SRC
Signaling pathways regulating pluripotency of stem cells	7.34E−02	BMP4, FGFR2, FGFR1, FGFR3, GRB2, IL6ST, SOX2, BMPR2, SMAD1, DVL1, MAPK1, MAPK14, WNT7A, KAT6A, BMPR1A	Influenza A	6.34E−02	AGFG1, MAP2K3, CREBBP, FASLG, NLRX1, TRIM25, NXF1, STAT2, VDAC1, PRKCB, ACTG1, TNFRSF1A, IKBKE, MAPK1, HNRNPUL1, MAPK14, CPSF4, IFNGR1, PIK3R2
Dorso-ventral axis formation	7.68E−02	MAPK1, ETS1, GRB2, SOS2, NOTCH4	PI3K-Akt signaling pathway	6.42E−02	PHLPP1, CSF3, FGFR2, FGFR1, YWHAZ, FGFR3, GRB2, PPP2R5C, FASLG, FOXO3, ITGB1, CDC37, SOS2, PRKAA1, MLST8, FGF1, GNG5, CHUK, PIK3R2, IL7, PKN2, COL5A3, YWHAE, DDIT4, MAPK1, CCND1, CDKN1B, YWHAH, GNB2, GNB1, VEGFA, YWHAQ, PDGFRA
Dopaminergic synapse	7.85E−02	GNAO1, GNAI2, PPP2R5C, GRIA4, PPP1CC, KCNJ3, PRKCB, PPP1CA, GNB2, GNB1, MAPK14, CALM3, GNAS, PLCB1	mRNA surveillance pathway	6.75E−02	PPP1CA, SMG5, NUDT21, PPP2R5C, CPSF6, PELO, CPSF4, ACIN1, PABPC1, NXF1, PPP1CC, RNGTT
Proteasome	8.41E−02	PSMC6, PSME1, PSMD11, PSMD4, PSME4, PSMD7, PSMB8	Viral carcinogenesis	7.71E−02	YWHAZ, GRB2, LOC684797, CREBBP, HDAC10, GTF2H4, YWHAE, SRF, PXN, SRC, HIST2H4, RT1-A2, CDC42, MAPK1, RT1-A1, CCND1, YWHAH, CDKN1B, HIST1H4B, YWHAQ, RBPJ, CHD4, TRAF3, PIK3R2
Endocrine and other factor-regulated calcium reabsorption	8.41E−02	DNM3, ATP1B1, AP2B1, GNAS, PLCB1, CLTC, PRKCB	Inositol phosphate metabolism	8.07E−02	INPP1, MTMR2, MTMR3, MTMR14, IMPAD1, CDIPT, PIK3C2A, PLCH2, PLCD4, SYNJ2
Protein export	8.63E−02	MGC109340, HSPA5, SRPRB, SEC61G, SEC61A2	Chemokine signaling pathway	8.23E−02	PARD3, GNAI2, GRB2, ADCY6, FOXO3, CX3CL1, PXN, SRC, STAT2, MAPK1, CDC42, GNB2, GNB1, SOS2, SHC1, RAP1B, GNG5, CHUK, PIK3R2
Alzheimer's disease	8.66E−02	ATP5D, NDUFA4, ATP5E, NDUFA8, APH1A, CYC1, IDE, PPP3R1, ATP5G2, ATP5G1, NDUFV3, MAPK1, TNFRSF1A, ATP2A2, MAPT, PSEN2, CALM3, PLCB1	Circadian entrainment	8.56E−02	RPS6KA5, MAPK1, GNAO1, GNB2, GNAI2, GNB1, ADCYAP1R1, ADCY6, GNAS, GRIA4, GNG5, PRKCB
Viral carcinogenesis	8.90E−02	YWHAZ, GRB2, IL6ST, LOC684797, CREBBP, GTF2H4, HDAC1L, MAPKAPK2, YWHAE, SRF, PXN, CDK2, SRC, RT1-A2, CDC42, MAPK1, RT1-A1, YWHAH, CDKN1B, YWHAQ, TRAF3, RT1-N2	Metabolic pathways	8.87E−02	LDHB, CDIPT, IMPAD1, HMGCR, CNDP2, PGAM1, ACSS1, TRAK2, IDUA, PLD3, PLD1, CRLS1, C1GALT1C1, GATM, QDPR, CHPT1, KDSR, PRPS2, ME1, CHKA, EXTL3, MGAT5B, GCLC, AHCY, SRM, CHKB, GLUD1, ATP6V1B2, ACAT2, CERS2, PLCH2, IDH2, B4GALT6, DNMT3A, MGAT4A, GCDH, B4GALT3, ACER1, ACACA, AK2, NDUFV3, GLS, AHCYL1, IDI1, NAT8L, ATP5D, ATP5E, NDST2, CYC1, MAN1B1, QARS, CKB, ACOX3, ACOT8, P4HA2, P4HA1, MCCC1, MGLL, SYNJ2, ATP5L, AGPAT4, PTDSS1, AGPAT3, PFKL, ACADS, PIK3C2A, PIGU, COQ5, MAN2A1, COQ3, MTMR14, PANK3, DGAT1, CHSY1, PLA2G3, DEGS1, INPP1, BCAT1, CYP2U1, GALNT2, GALNT1, GALNT7, UGDH, ATP5G2, ATP5G1, MTMR2, MTMR3, MTHFR, STT3A, AKR1A1, DGKE, DGKG, PLCD4, TSTA3, ACSL3, HPGDS, HSD17B7, GALNT14, B4GALNT1, HSD17B8, NDUFA4, DLST, NDUFA8, ACLY, POLR3C
TNF signaling pathway	9.93E−02	RPS6KA5, VCAM1, MAPK1, TNFRSF1A, TNFRSF1B, DNM1L, MAP2K3, MAPK14, CX3CL1, TAB2, CHUK, TRAF3	TNF signaling pathway	8.90E−02	RPS6KA5, VCAM1, TNFRSF1A, MAPK1, TNFRSF1B, DNM1L, MAPK14, MAP2K3, CX3CL1, TAB2, CHUK, TRAF3, PIK3R2
			Long-term potentiation	9.12E−02	MAPK1, PPP1CA, RPS6KA1, ARAF, CREBBP, PPP3R1, RAP1B, PPP1CC, PRKCB
			Melanogenesis	9.57E−02	MAPK1, EDNRB, GNAO1, GNAI2, ADCY6, MITF, CREBBP, GNAS, TCF7L2, WNT7A, DVL1, PRKCB

**Figure 4:**
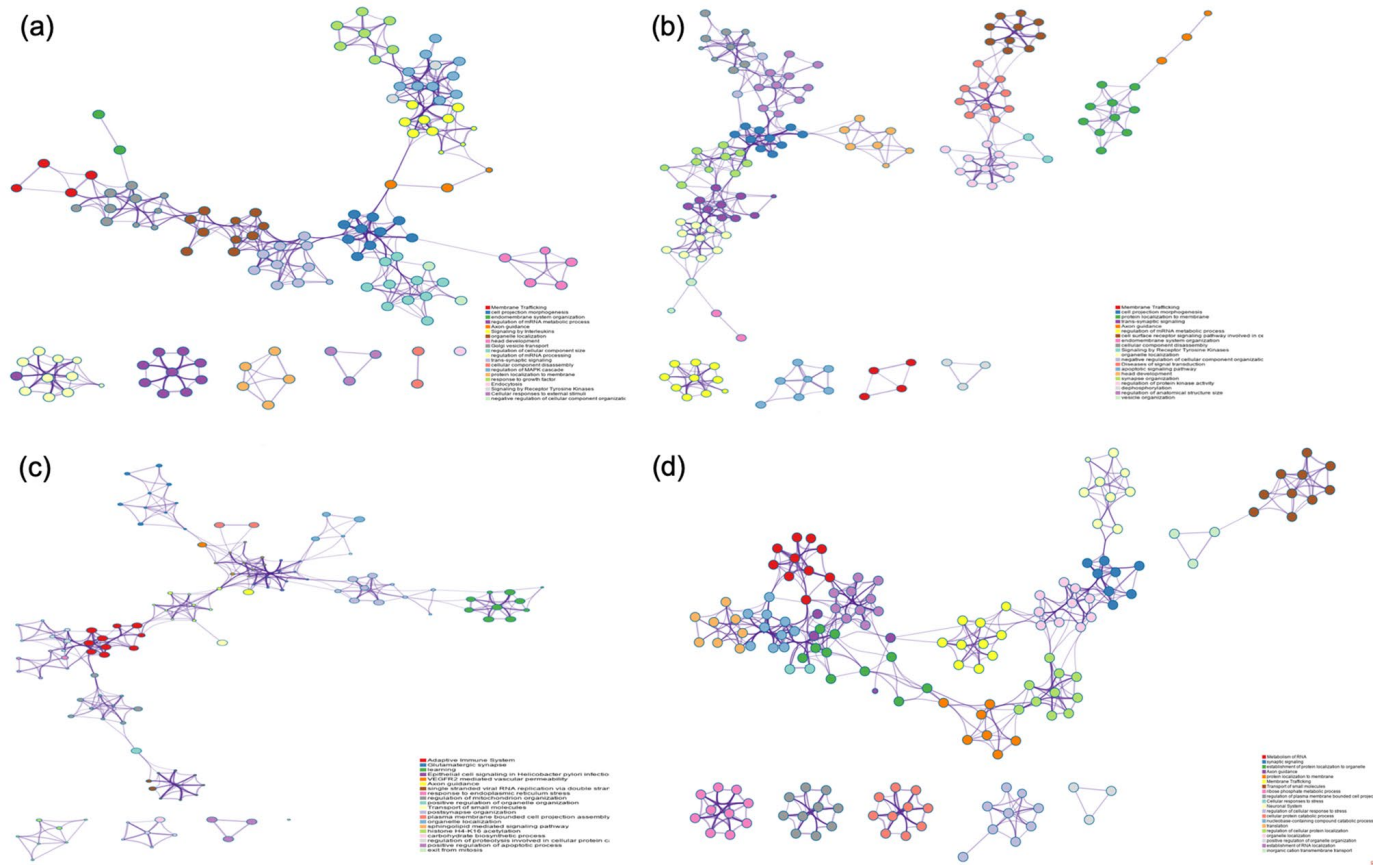
Metascape enrichment network visualization showing the intra-cluster and inter-cluster similarities of enriched terms, up to ten terms per cluster. Axon guidance pathway interaction with other pathways have been shown. (**a**) Upregulation following perinatal alcohol exposure compared to saline control. (**b**) Upregulation following perinatal nicotine–alcohol exposure compared to saline control. (**c**) Downregulation following perinatal alcohol exposure compared to saline control. Cluster annotations are shown in color code. (**d**) Upregulation following perinatal nicotine–alcohol exposure compared to the alcohol exposure.

Additionally, we used Metascape^58^ enrichment network visualization to show the intra-cluster and inter-cluster similarities of enriched terms and to predict the interactions among biological pathways using the significant DEGs following each treatment^58^. Metascape uses human targets as a default and analyzes different model organisms, giving us a most comprehensive human-centric database^58^. Figure 4 shows our DEG lists analyzed as human species, allowing us to predict affected downstream pathways and protein complexes within the human genome. This analysis confirmed the axon guidance pathway was affected in each treatment group. After perinatal alcohol treatment within our upregulated DEG list, axon guidance pathway was interacting with signaling by interleukins and cell projection morphogenesis pathways (Fig. 4a). Following perinatal nicotine–alcohol, our upregulated DEG lists showed axon guidance pathway showed interaction with protein localization to membrane (Fig. 4b). After perinatal alcohol treatment, our downregulated DEG lists showed that axon guidance pathway was interacting with VEGFR2 mediated vascular permeability (Fig. 4c). After perinatal nicotine–alcohol treatment when compared to the alcohol treatment group, our upregulated DEG lists showed that axon guidance pathway interacts with membrane trafficking, establishment of protein localization to organelle, and cellular responses to stress (Fig. 4d).

## Discussion

Recent statistics show that 7.2 and 11.5% of mothers use nicotine and alcohol during pregnancy, respectively^15,16^. The effects of drug use are lifelong and can be severe including birth complications and developmental disabilities^14^. In this study, we focused on identifying large scale miRNA and gene expression profiling in the DA neurons of the VTA following perinatal alcohol or nicotine–alcohol exposure in male rat pups. The time frame of exposure in rats was equivalent to the three trimesters of human pregnancy. In our recent study, we have found the expression of many miRNAs and mRNAs to be significantly altered following perinatal nicotine exposure in the VTA DA neurons of rat pups^31^. Among the biological pathways affected, dopaminergic synapse pathway, nicotine addiction, as well as neurotrophin signaling pathway were enriched compared to control. This study encouraged us to investigate the influence of maternal alcohol intake and exposure and expand this study to include maternal alcohol and nicotine combined exposure. Subsequently, we used a similar protocol as in our previous study^31^ which investigated perinatal nicotine exposure alone compared to saline. For the alcohol group, pregnant mothers were fed a Lieber–DeCarli ethanol diet. This widely practiced and established method provides high protein ethanol diet to the pregnant mother without inducing stress or compromising the mother’s health^50,59^. Regarding the nicotine–alcohol group, we used our established nicotine animal model while feeding them the liquid ethanol diet throughout the 4 weeks of gestational exposure^24,30,35^. Following our differential expression analysis of the alcohol-treated group, we found 1,257 DEGs to be upregulated, and 330 DEGs downregulated. Our nicotine–alcohol treatment differential expression analysis identified 1,771 DEGs upregulated, and 269 DEGs downregulated. Among the microRNAs, there were 51 DEmiRs that were upregulated and 39 DEmiRs that were downregulated following perinatal alcohol exposure. Following perinatal nicotine–alcohol exposure, 51 DEmiRs were upregulated and 41 DEmiRs were downregulated. Validated and predicted correlation between our DEmiRs and DEGs target pairs was performed and analyzed following each treatment group to identify possible miRNA to gene pairs and putative miRNA targets resulting from perinatal alcohol or nicotine–alcohol exposure. To better interpret the function of these DEGs within biological processes during development, pathway enrichment analysis using DAVID was performed. Finally, we analyzed the DEG lists following perinatal alcohol and nicotine–alcohol exposure groups through Metascape to further study the interactions between enriched pathways, and apply our findings to genes within the human genome.

After analyzing the predicted and validated miRNA–gene target pairs, we found the miRNA miR-30b had the greatest number of connections with the target genes in both alcohol and nicotine–alcohol treatment. This suggests the potential of miR-30b in controlling the expression of many genes involved in different biological processes during neurodevelopment following nicotine and/or alcohol exposure. In our study, following the alcohol treatment miR-30b was predicted to target the DEG GNAI2, a G protein subunit alpha i2. Recently, using whole‐exome sequencing analysis, a de novo heterozygous missense mutation in the GNAI2 gene was found in an individual with periventricular nodular heterotopia and intellectual disability^60^. Additionally, in vivo studies conducted with GNAI2‐knockdown mice determined a lack of social interaction, recognition and increased anxiety in these mice^60^. Together, these studies suggest an important role for GNAI2 in healthy brain development. Moreover, GNAI2 has been linked to long-term depression in the neurodevelopment^61^. Another predicted DEG that was targeted by miR-30b was COTL1, which is a coactosin-like F-actin binding protein 1. COTL1 competes with cofilin to bind to F-actin, and involves neuronal migration^62^. Early- and late-born cortical neurons display distinct migratory behaviors^63^. A study conducted by Li et al. revealed that COTL1 overexpression impaired migration of both early- and late-born neurons during mouse corticogenesis, which suggests COTL1 was required for both somal translocation and locomotion, which can further impact cognitive/emotional/behavioral development^64^. Additionally, a study conducted by Bosch et al. shows that miR-30b was downregulated in the VTA of methamphetamine self-administering rats^65^. Their results indicate the importance of miR-30b across different addictive substances, suggesting this miRNA potentially plays a role in drug reinforcement and neuroplasticity^65^.

Within the nicotine–alcohol group, miR-30b was predicted to target GNAI2, as well as BNIP3L, which is a BCL2 interacting protein 3 like, and part of the Bcl2 apoptotic pathway. BNIP3L induces apoptosis by directly targeting the mitochondria and causing apoptosis-associated mitochondrial changes^66^. It has been shown that increased cell death in the nervous system could lead to several neurodegenerative pathologies^67^. Induced expression of BNIP3L promoted its localization to the mitochondria, triggered a loss of membrane potential, and increased reactive oxygen species production, which ultimately leads to cell death^68^. Therefore, the BCL2 family has been an important focus of neuroscientific interest due to its potential influence on neurodegenerative pathologies. Overall, these predicted targets suggest alterations during the neurodevelopmental processes at the cellular level following both alcohol and/or nicotine exposure through modulation of genes associated with neural migration, neurodevelopment and apoptosis.

After analyzing the predicted and validated miRNA–gene target pairs in the nicotine–alcohol treatment group compared to the alcohol only group, we found the miRNA miR-26b had the greatest number of connections with the target genes. miR-26b has been previously shown to play a role in the basic mechanisms of brain neuroplasticity, stress response and in the pathogenetic mechanisms of several neuropsychiatric diseases^69^. Within the nicotine–alcohol vs. alcohol treatment group, miR-26b was predicted to target NXPE3, which is a neurexophilin family of neuropeptide-like glycoproteins promoting adhesion between dendrites and axons^70^. Defects in this may result in brain abnormalities as NXPE3 has been linked to epilepsy^71,72^. This suggests alterations in neural migration and connections during the neurodevelopmental processes following nicotine–alcohol perinatal exposure compared to the alcohol perinatal exposure group at the cellular level.

Following our differential expression analysis and miRNA–gene target pairs, we further analyzed the enriched KEGG pathways to understand putative processes regulated by perinatal alcohol and nicotine–alcohol exposures. We found that glutamatergic synapse and axonal guidance pathways following perinatal alcohol and perinatal nicotine–alcohol exposure in the DA neurons of the VTA were enriched. Glutamate is the major excitatory neurotransmitter in the mammalian brain accounting for approximately 70% of synaptic transmission within the central nervous system^73–75^. Glutamate pathways are linked to many other neurotransmitter pathways as glutamate receptors are found throughout the brain and spinal cord in neurons and glia^76^. A subset of DA neurons in the VTA co-release DA and glutamate to the NAc and are believed to play a role in behavioral activation following stimulants, illustrating a role in drug addiction^77–79^. This could partly explain the enriched glutamatergic synapse pathway as alcohol directly activates the DA, playing an essential role in neurodevelopment^80^. Glutamatergic synapse pathway was enriched following both treatments perinatally compared to control (p < 0.001 for alcohol, p < 0.01 for nicotine–alcohol). Among the DEmiRs within the glutamatergic synapse pathway following perinatal alcohol exposure, miR-410-3p (p value of < 0.05) and miR-298-5p (p value of < 0.01) had further connections. miR-410-3p was predicted to target and inhibit TRPC1, which encodes a membrane protein forming a non-selective channel permeable to calcium and other cations^81^. TRPC1 has been shown to affect the group I metabotropic glutamate receptors pathway and auditory signal processing at the midbrain level^82^. Additionally, Xiong et al. showed that low miR-410-3p expression was associated with the chemotherapy drug, gemcitabine resistance in human pancreatic cancer xenograft tumor tissues and pancreatic ductal adenocarcinoma (PDAC) cells as well as poor prognosis in PDAC patients. TRPC1 as one of the potential targets of miR-410-3p was also significantly affected by the miR-410-3p expression modifications^83^. This data may suggest that the glutamatergic system plays a role in non-neuronal tissues including tumor biology^84^. miR-298-5p was predicted to target and inhibit HOMER3 which encodes a postsynaptic density scaffolding protein^81^, which in part regulates signal transduction and maintains extracellular glutamate levels in corticolimbic brain regions^85^. A review study by Szumlinski et al. suggests members of the Homer protein family regulates drug-induced neuroplasticity through glutamate receptor trafficking^85^.

The glutamatergic synapse pathway following perinatal nicotine–alcohol exposure shows a more complex pathway with several intracellular regulatory points. Among the many DEmiRs, miR-449a (p value of < 0.05) was found to significantly inhibit genes within different clusters. miR-449a showed a strong specificity for lung, testis, and adenocarcinoma tissues and to be involved in the development of carcinoma by being a potential inducer of cell death, cell-cycle arrest, and/or cell differentiation^86^. Our data showed that miR-449a was predicted to target PRKCB, which further connects and activates MAPK3. MAPK3 is known to be involved in the control of cell proliferation, cell differentiation, and cell survival^87^. Moreover, pathways and functional linkages in the large set of genes were associated with autism spectrum disorders (ASD). Based on the common ASD genes in the MAPK (MAPK3) and calcium signaling pathways (PRKCB), the overlapping function of these two pathways in ASDs were narrowed down to voltage-gated calcium channels and calcium activated PKC^88^. DEmiRs miR-290 (p value of < 0.05) and miR-7b (p value of < 0.05) also targeted the same gene cluster as miR-449a, but were specifically connected to the genes MAPK1 and Phospholipase D1(PLD1), respectively. PLD1 has been shown to negatively affect glutamate function under oxidative stress conditions, which highlighted the role of PLDs in glutamate transporter regulation in the synaptic endings exposed to oxidative injury^89^. miR-30b-5p was predicted to have the greatest number of connections downstream and was also differentially expressed in this pathway, affecting different gene clusters, predicted to connect and inhibit Shank3, which plays a role in the function of synapses, ensuring signals are received by the postsynaptic neuron in the brain^90^. The glutamatergic synapse pathway was looked at following perinatal nicotine–alcohol exposure compared to perinatal alcohol exposure group (p > 0.05) for comparison.

During the development of the nervous system, neurons extend their axons to reach their targets, forming functional circuits. These circuits are the basis of neural function and their faulty assembly can result in disorders of the nervous system^91^. A study conducted by Lindsley et al. suggested that ethanol disrupted the way axons respond to guidance cues effecting axon growth and elongation^92^. Our results supported this data as we found the axonal guidance pathway to be enriched following both treatments perinatally compared to control (p < 0.01 for alcohol, p < 0.01 for nicotine–alcohol). The axonal guidance pathway following perinatal alcohol exposure had many DEmiRs, among which mir-15b-5p had a significant predicted interaction with a p value of < 0.001 targeting DEG GNAI1. Additionally, Lewohl et al. reported miR-15b to be up-regulated in the prefrontal cortex of human alcoholics^93^. This data may suggest possible similarities in the altered expression of genes involved in the neural networks of an addicted brain and a developing brain exposed to alcohol. miR-495 (p ≤ 0.02) was predicted to target and inhibit two gene from different gene clusters, KRAS, part of the RAS oncogene family, involved in cell growth, maturation, and death^94^, and Wnt4, which plays critical roles in many biological processes including embryonic development^95^. KRAS was further predicted to activate MAPK1/3, playing a role in regulating multiple physiological processes including mitosis, cell differentiation, and cell survival. The axon guidance pathway following perinatal nicotine–alcohol exposure compared to perinatal alcohol exposure group was not significant (p > 0.05).

Following perinatal nicotine–alcohol exposure, the axonal guidance pathway showed the significantly differentially downregulated mir-466b-5p (p value of $\le$ 0.0001) inhibiting Integrin Subunit Beta 1 (ITGB1), which in turn activated UNC5B and PTK2. UNC5B encodes a protein that is part of the dependence receptors (DPRs) proteins, which are said to be involved in embryogenesis and cancer progression^96–99^. PTK2 gene activation is said to be a crucial step early on in the cell growth and intracellular signal transduction pathways^100,101^. miR-34c-5p (p < 0.05) was predicted to target and inhibit SLIT1, which was differentially upregulated and predicted to connect and activate SLIT2, SRC-like kinase FYN and ROBO1 genes. FYN has been implicated in the control of cell growth^102^ and has been linked to cancer pathogenesis^103^. The Slit family of secreted glycoproteins were originally identified in the nervous system functioning as axon guidance cues and branching factors during development regulating neuronal axon guidance, neuronal migration, cell proliferation and cell motility through its binding to Robo receptors^104,105^. The overall results suggest neuronal development to be highly modulated at many putative points resulting in alterations within many biological processes crucial to development such as cellular growth, differentiation, signal transduction, synapses, and cell survival.

Pathways in cancer, Wnt signaling pathway, long-term potentiation, Huntington’s disease, and Parkinson’s disease were among the many significantly enriched KEGG pathways following the nicotine–alcohol perinatal exposure group compared to the alcohol perinatal exposure group. Prenatal alcohol exposure has been shown in previous studies to disrupt Wnt signaling pathway and has been a determinant of FASD^106,107^. In the central nervous system, Wnt signaling is known to modulate neuronal proliferation, migration, adhesion, differentiation, and axon outgrowth^108–112^. FASD have also been linked to abnormal neuronal plasticity responsible for normal wiring of the brain and involved in learning and memory^113^. Many studies have shown this by corresponding disruptions in long term potentiation with acute or chronic perinatal alcohol exposure^114–117^.

We further looked at the regulation and connection of the enriched axon guidance pathway with other biological pathways and protein complexes by using Metascape, which converts the given gene lists from rat into human Entrez gene IDs. Following this analysis, axon guidance from our upregulated DEGs following perinatal alcohol exposure was shown to directly connect to cell projection morphogenesis. Cell morphogenesis is the process in which the neurons are generated, organized and targeted to a specific site in response to attractive or repulsive cues^118^. Axonogenesis refers to the morphogenesis of shape or form of the developing axon, which carries efferent action potentials from the cell body towards target cells. Axon guidance is one of the important parts of the axonogenesis during which the migration of an axon is directed to a specific target. This data also confirmed that an upregulation in these two processes closely linked them to each other. Axon guidance from our downregulated DEGs following perinatal alcohol exposure was connected to VEGFR2 mediated vascular permeability. Alcohol-induced premature retraction of the radial glia in the deep cortex and alcohol-induced retardation in neuronal migration have been already shown in the literature^119–122^. Considering VEGF is a chemoattractant for commissural axons in vitro and in vivo^123^ and that growing axons are guided to their targets by attractive and repulsive cues, it is very likely that if there is a downregulation in the DEGs due to alcohol exposure, these two pathways will influence each other. Axon guidance from our upregulated DEGs following perinatal nicotine–alcohol exposure was directly connected to protein localization to membrane, which could suggest a broad modulation among many pathways affecting protein function and translation. Axon guidance was also connected to membrane trafficking, establishment of protein localization to organelle, and cellular responses to stress.

In conclusion, we have conducted a large-scale miRNome and transciptome study following perinatal alcohol and nicotine–alcohol exposure in the DA neurons of the VTA of male rat pups. We have investigated transcriptional and post transcriptional alterations and putative regulatory points within neurodevelopmental pathways in the postnatal brain and possible disruptions within biological pathways systemically. We identified enriched biological pathways following each treatment, and downstream gene network interactions between these significant pathways within the human genome. Our study suggested Glutamatergic synapse and axon guidance pathways to be significantly enriched and many miRNAs and genes were altered following nicotine or alcohol-nicotine exposure perinatally. Cell growth, proliferation, neuronal migration, neuronal axon guidance, and cell survival cues were among pathways in which many genes and miRNAs were significantly altered in response to perinatal alcohol or nicotine–alcohol exposure. Our nicotine–alcohol exposure compared to saline group showed the nicotine addiction pathway was enriched, which was also seen in our previous nicotine only study, comparing perinatal nicotine exposure to saline^31^. Additionally, the glutamatergic synapse pathway was enriched in all groups (nicotine, alcohol, and combined nicotine–alcohol) when compared to saline^31^. Although, our results using rat model of 3‐trimester gestational exposure to both alcohol and nicotine indicate that perinatal alcohol and nicotine exposure alters the expression of miRNAs and genes in infant rats, it should be noted that it is important to validate these models by translating them into human studies. Variable factors in this study include the alcohol intake, nicotine doses and body weight variations across dams which could potentially affect the results. Additionally, we limited our initial study to only include male pups. Such a limitation requires further studies to be conducted using female pups to explore gender differences following perinatal alcohol and/or nicotine exposure. Further studies need to be conducted to better understand the systemic putative pathway regulation points at a molecular level from gene expression profiling to protein translation, as well as to investigate therapeutic approaches that target disorders associated with gestational addictive substance exposure such as FASDs.

## Materials and methods

### Animal treatment

All experimental protocols and surgical procedures were approved by the Institutional Animal Care and Use Committee (IACUC) and the University of Houston Animal Care Operations (ACO) and were performed in accordance with accepted guidelines and regulations. Sprague Dawley (SD) rats are one of the most commonly used outbred rat strains for biomedical research, providing 95% or greater accuracy on timed-pregnant gestation and ideal for safety and efficacy testing, aging, behavior, reproduction and surgical modifications^124,125^. Pregnant female SD rats purchased from Charles River (Charles River, Wilmington, MA, USA) were housed in the animal facility and were maintained at 22 ± 2 °C with 65% humidity, on a 12-h light/12-h dark cycle. The animal treatment method has been further detailed in Keller et al.^31,32^. Briefly, rats were acclimated to the animal facility upon arrival for 72 h before the subcutaneous insertion of an osmotic pump (Alzet, Cupertino, CA, USA) containing either nicotine hydrogen tartrate (Sigma-Aldrich, St. Louis, MO, USA), which released nicotine at a rate of 6 mg/kg/day in order to simulate the nicotine plasma level found in moderate smokers^31,32^, or an equal volume of saline for control^126^. Liquid diet containing 36% kcal from ethanol F1265SP or F1264SP control, purchased from Lieber-DeCarli (Bio-Serv, Flemington, NJ, USA) was placed in Richter feeding tubes (Bio-Serv), and was gradually introduced to the pregnant mothers based on the protocol provided from Bio-Serv. This liquid diet model has reliably produced blood alcohol concentrations (BACs) between 80 and 180 mg/dl in rats, which are accompanied by neurological deficits similar to what is observed in children with FASD^50,127–133^. Pregnant dams on average consumed around 80–100 ml of liquid diet per day. A total of 12 dams were used in this study. 4 were used for saline, 4 for alcohol, and 4 for nicotine–alcohol. Pups would be exposed to either alcohol or nicotine and alcohol for four weeks starting from gestational day 6 to postnatal day 14, equivalent to the three trimesters in human gestation during which rapid brain growth and synaptogenesis occur^38,49^. The central nervous system development of a rat at postnatal age of 7–14 days is suggested to correspond approximately to the human brain at term^134^. The male pups collected from P10 to P14 were all considered as “infants”, therefore, they were pooled from each litter for this study^135,136^. Supplementary Figure S1 summarizes the overall methodology.

Perinatal drug exposure including nicotine, alcohol and combined exposure has been known to produce changes in a sex dependent manner^137–140^. Previous studies considering early-life exposure to these drugs have been done predominantly on male subjects or rodents^141^. Perinatal nicotine exposure has been shown to have more significant deleterious effects on the cholinergic and serotonergic markers in males than females^140,142^. Drug reward is also shown to be altered by prenatal nicotine exposure with increased preference for nicotine in males than females^140,143^. For this reason, males only were examined in this study. Male and female rat pups were distinguished by a larger genital papilla and longer ano-genital distance in male vs. female pups^137^. Postnatal 10 to postnatal 14-day old male pups were anesthetized using isoflurane gas before decapitation on a VT1200 semiautomatic vibrating blade microtome (Leica, Nussloch, Eisfeld, Germany). 1 mm thick horizontal brain slices containing the VTA were sliced and 1 mm biopsy punch (Integra Miltex, VWR, Radnor, PA, USA) was used to collect the VTA bilaterally. Brain punches from 4 to 7 pups from each litter were pooled and placed on ice in Hibernate A (Gibco, Thermo Fisher Scientific, USA) to preserve and maintain cell viability. Brain punches were pooled for each litter and a total of four samples, 16–28 pups/sample for each alcohol, nicotine–alcohol, and saline treated groups were collected, processed, and analyzed, for a total of 48–84 pups used in this study.

### Brain slice preparations and FACS cell sorting

Brain tissue punches containing the VTA were collected and dissociated into a single cell suspension before sorting using FACS as previously reported by Guez-Barber et al.^144^. Briefly, collected tissue punches were dissociated in Accutase (Gibco, Thermo Fisher Scientific, Waltham, MA, USA) and shaken at 4 ℃ for 30 min. Cells were centrifuged and pelleted at 425×*g* and resuspended in Hiberante A medium (Gibco). Cell aggregates were then further dissociated through gentle pipetting with increasingly smaller pipette tips; the supernatant containing single cells were collected. Cellular debris was then removed using serial filtration. First, the cell suspension was run through a pre-wetted 100 µm cell strainer and then through a pre-wetted 40 µm cell strainer while on ice. Further removal of small cellular debris was done by density centrifugation. The cell suspension was added to the top of a three-density gradient which was made using Percoll (GEHealthcare, VWR, USA) and centrifuged for 3 min at 430×*g*. After centrifugation, the cloudy top layer, which contained cellular debris was removed. The remaining cell suspension was pelleted through centrifugation at 550×*g* for 5 min.

To fix cells for immunolabeling, cells were resuspended in 1 ml of Hibernate A and 1 ml of cold absolute ethanol, gently vortexed and stored on ice for 15 min. Cells were incubated and labelled with conjugated primary antibodies neuronal marker, NeuN/Alexa Fluor 488 (NeuN/AF488, ab190195, Abcam, Cambridge, MA, USA), and tyrosine hydroxylase/phycoerythrin (TH/PE, ab209921, Abcam, Cambridge, MA, USA) and rotated for 30 min at 4 ℃. Cells were then washed with PBS and centrifuged at 950×*g* for 3 min before they were resuspended in PBS. Flow cytometry was performed on an (LSR II) FACS Aria (BD Biosciences) instrument and analyzed using FlowJo software at the Baylor College of Medicine Cytometry and Cell Sorting Core (One Baylor Plaza, Houston, TX, USA). DA neurons with intact nuclei were labelled and sorted based on their positive double staining of NeuN and TH, populations were distinguished by their forward and side scatter, and two-parameter density plots were measured with gating parameters set at around 10^3^ for NeuN FITC and 10^4^ for TH-PE expression.

### RNA extraction

Following FACS, cells were pelleted by centrifugation at 2,650×*g* for 8 min at 18 ℃ and total RNA was extracted using miRNeasy Micro Kit (Qiagen, Hilden, Germany) including DNAse treatment following manufacturer’s instructions. A NanoDrop 2000 spectrophotometer (Thermo Fisher Scientific) was used to check the RNA purity and quantity according to the optical density (OD) of each sample at 260 nm and 280 nm. Only samples with a 260/280 ratio of 1.9 or greater were used in experiments.

### Microarray preparation, labeling, and hybridization for gene and microRNA expression profiling

All kits that were used for both mRNA and miRNA gene expression analysis were purchased from Agilent (Santa Clara, CA, USA) unless stated otherwise. SurePrint G3 Rat Gene Expression v2 8 × 60 K microarray (ID: 074036) with 30,584 unique genes was used for the mRNA expression profiling using 25 ng of total RNA. Samples were prepared and labeled according to manufacturer’s instructions for the One-Color Microarray-Based Gene Expression Analysis using the One-Color Low Input Quick Amp Labeling kit with RNA Spike-Ins. The labeled amplified complimentary RNA was then purified using the RNeasy Mini Kit (Qiagen) and quantified using a NanoDrop 2000 spectrophotometer. The cRNA yield and specificity was then calculated according to manufacturer’s instructions. Microarrays were then hybridized for 17 h at 65 ℃ using the Gene Expression Hybridization kit according to manufacturer’s instructions. miRNA expression profiling was performed using an 8 × 15 K Rat miRNA Microarray, Release 21.0 (ID: 070154) containing 758 mature miRNAs. A starting quantity of 100 ng total RNA containing miRNAs was used following manufacturer’s instructions of the MicroRNA Microarray System with miRNA Complete Labeling and Hyb Kit. Purification of the labeled RNA was done on a Micro Bio-Spin P-6 gel column (Bio-Rad, Hercules, CA, USA) following manufacturer’s instructions. Samples were then dried using a vacuum concentrator with heater at 50 ℃ and hybridized at 55 ℃ for 20 h. Slides were then washed following manufacturer’s instructions using Gene Expression Wash Buffers containing Triton X-102. Both gene and miRNA expression slides were scanned following hybridization using a G4900DA SureScan Microarray Scanner. The raw microarray dataset was then collected from the resulting images using the Feature Extraction Software v12.0.1.

### Data analysis

Analysis and comprehension of the genomic data was performed using Bioconductor packages in R version 3.6.1^145^. The limma^146^ package was used for mRNA expression data loading and following procedures including the removal of outliers, background correction and quantile normalization were performed. We defined low expression as any intensity less than 75% brighter than 90% intensity of negative controls. Replicates were removed and a total of 11,791 genes were collected for analysis. Fold change and standard errors were calculated using lmFit function, which fits multiple linear models by weighted least squares. Standard errors were moderated using eBayes function, which computes log-odds of differential expression by an empirical Bayes model. DEGs were identified using a series of p values (i.e. 0.05, 0.01, 0.001), with the minimum log2 fold change > 1 and adjusted using the BH method. DEmiRS were identified using a series of p values (i.e. 0.05, 0.01, 0.001), with the minimum log2 fold change > 0.5 and adjusted using the BH method.

The AgiMicroRna^147^ package was used for the miRNA expression data loading and processing. Raw data was loaded using readMicroRnaAFE function, and preprocessed using rmaMicroRna function, which implements the robust multi-array average (RMA) algorithm. Data was then filtered using filterMicroRna function and only detected genes which were expressed in at least 50% of samples, with higher intensity than the mean value of negative control + 1.5 standard deviations, were collected for analysis. After preprocessing, 332 miRNAs remained. Similar to mRNA data processing, linear model was fitted to the miRNA expression data and moderated statistics were calculated using eBayes. Differential expression was identified using a series of p values (i.e. 0.05, 0.01, 0.001) and adjusted using the BH method.

### Comprehensive analysis of differentially expressed gene and microRNAs

MultiMiR^51^ package was used to identify the predicted and validated miRNA–mRNA pairs based on the inversely correlated regulation between miRNA and target genes. miRNA–mRNA pairs were identified using a series of p values (i.e. 0.05, 0.01, 0.001) and labeled accordingly. Network visualization was conducted using Gephi^148^, which is an open-source software for network visualization and analysis. Three colors were assigned to edges with different p values (i.e. green for p = 0.05, blue for p = 0.01, and red for p = 0.001). The color blue was then assigned to all the miRNAs and yellow for all the mRNAs. The size of the vertex was determined based on an out-degree, which represents the number of edges formed by an incident. A topology filter was then applied to filter out the vertex, which do not have any connections. Functional enrichment analysis was performed on our integrated network of DEGs and DEmiRS using DAVID version 6.8^52,53^. Enriched KEGG pathways were then identified and further analyzed using KEGGgraph^57^. Metascape^58^ was used on our DEG list following alcohol and nicotine–alcohol treatment to show enriched pathways as a network, therefore further understand relationships among the enriched pathways and their correlation to each other through their downstream connections.

## Supplementary information

Supplementary Information
