# Supplementary material for: Investigating the influence of perinatal nicotine and alcohol exposure on the genetic profiles of dopaminergic neurons in the VTA using miRNA–mRNA analysis
Source: Sci Rep. 2020 Sep 14;10:15016. doi: 10.1038/s41598-020-71875-1 (PMC7490691; doi:10.1038/s41598-020-71875-1)

**Investigating the influence of perinatal nicotine and alcohol exposure on the genetic profiles of dopaminergic neurons in the VTA using mRNA-miRNA analysis**

Tina Kazemi^1^, Shuyan Huang^1^, Naze G. Avci^1^, Charlotte Mae K. Waits^1^, Yasemin M. Akay^1^, and Metin Akay^1*^

^1^University of Houston, Department of Biomedical Engineering, Houston, TX, 77204, USA

^*^Corresponding author: [maka](mailto:makay@uh.edu)[y@uh.edu](mailto:y@uh.edu)

**
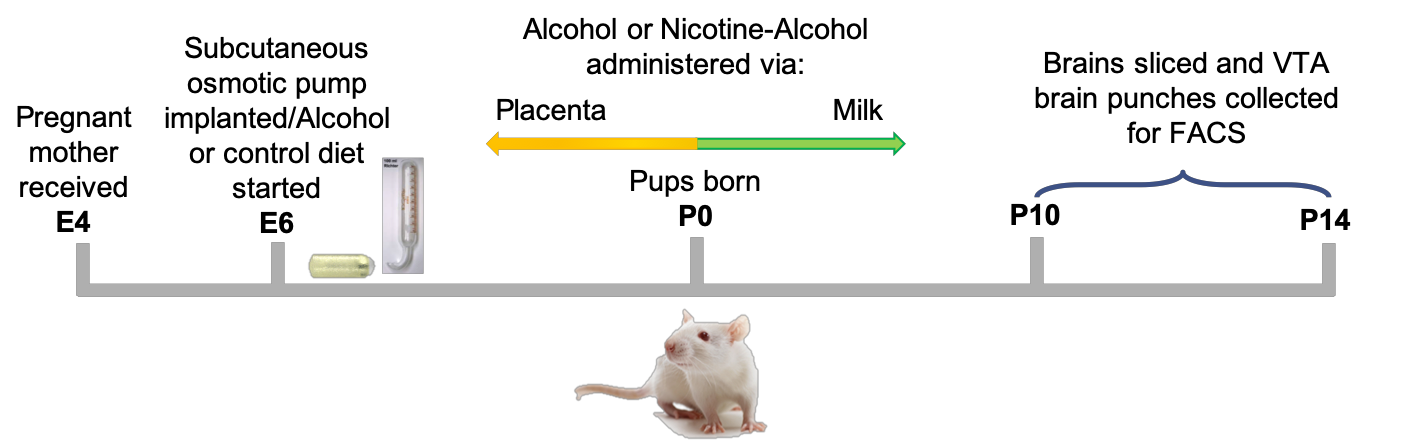
**

**Supplementary Figure 1. Summary of the experimental timeline.**

**Supplementary Table 1. KEGG pathways** enriched by (**a**) down and (**b**) upregulated DEGs and the corresponding genes identified in pathway analysis following perinatal nicotine-alcohol exposure compared to alcohol exposure.


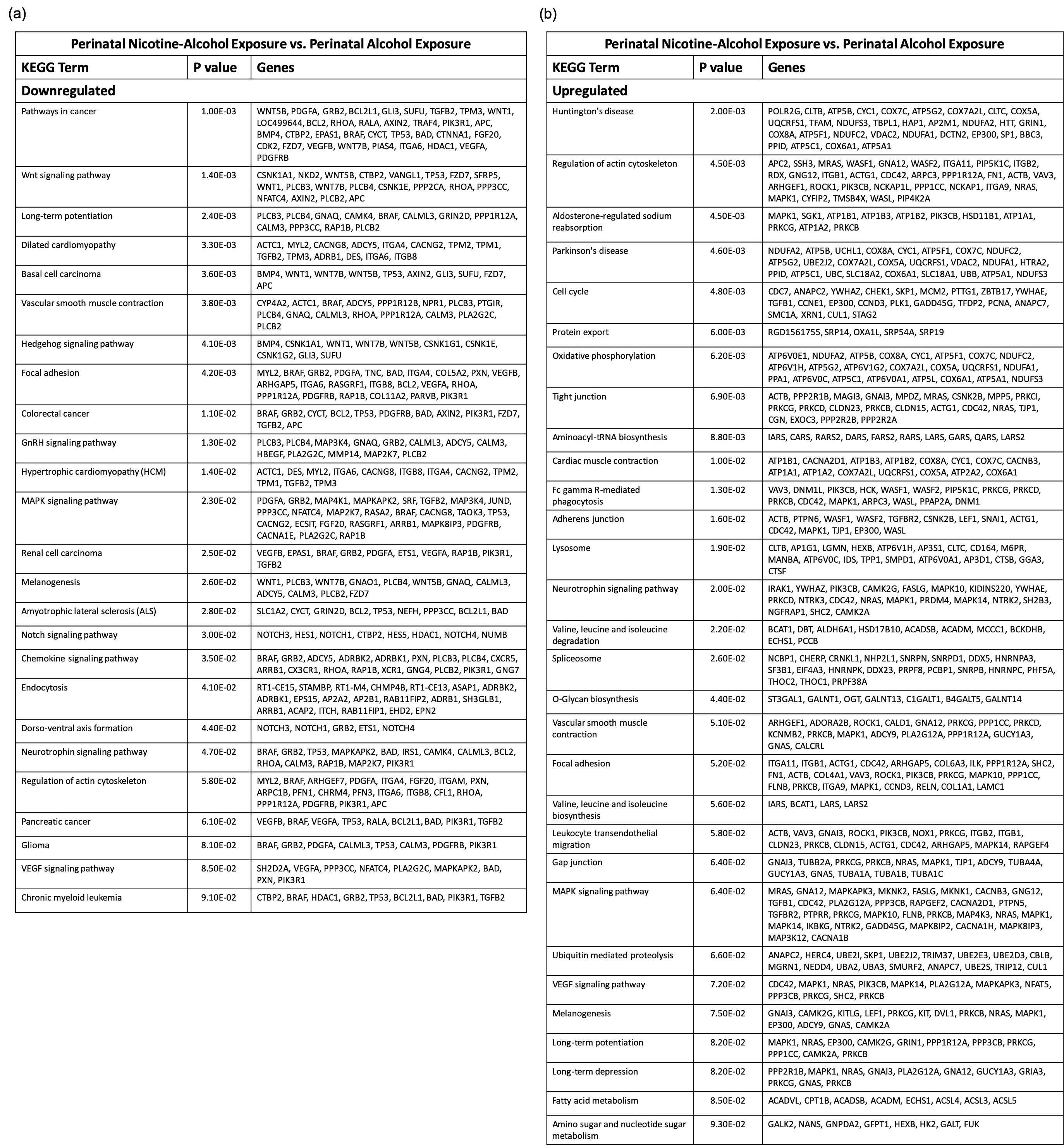

Supplement: Supplementary file 1 — Supplementary Information [file 41598_2020_71875_MOESM1_ESM.docx]
